# Traumatic Brain Injury and Risk of Incident Comorbidities

**DOI:** 10.1001/jamanetworkopen.2024.50499

**Published:** 2024-12-12

**Authors:** Cathra Halabi, Saef Izzy, Anthony M. DiGiorgio, Hunter Mills, Farid Radmanesh, John K. Yue, Habibeh Ashouri Choshali, Gundolf Schenk, Sharat Israni, Ross Zafonte, Geoffrey T. Manley

**Affiliations:** 1 Department of Neurology, University of California, San Francisco; 2 Weill Institute for Neurosciences, University of California, San Francisco; 3 Department of Neurology, Brigham and Women’s Hospital, Boston, Massachusetts; 4 Harvard Medical School, Boston, Massachusetts; 5 The Football Players Health Study at Harvard University, Boston, Massachusetts; 6 Department of Neurological Surgery, University of California, San Francisco; 7 Institute for Health Policy Studies, University of California, San Francisco; 8 Bakar Computational Health Sciences Institute, University of California, San Francisco; 9 Division of Neurocritical Care, Department of Neurology, University of New Mexico, Albuquerque; 10 Department of Physical Medicine and Rehabilitation, Massachusetts General Hospital, Brigham and Women’s Hospital, Boston; 11 Spaulding Rehabilitation Hospital, Charlestown, Massachusetts

## Abstract

**Importance:**

Traumatic brain injury (TBI) is associated with chronic medical conditions. Evidence from diverse clinical administrative datasets may improve care delivery.

**Objective:**

To characterize post-TBI risk of incident neuropsychiatric and medical conditions in a California health care system administrative database and validate findings from a Massachusetts dataset.

**Design, Setting, and Participants:**

In this cohort study, prospective longitudinal cohorts using data from 5 University of California health care settings between 2013 and 2022 were studied. Patients aged 18 years and older with mild (mTBI) or moderate to severe TBI (msTBI) were included. Unexposed individuals were propensity matched by age, race and ethnicity, sex, University of California site, insurance coverage, area deprivation index (ADI) score, and duration from index date to most recent clinical encounter. Patients with study comorbidities of interest before the index date were excluded. Data were analyzed August to October 2024.

**Exposure:**

TBI.

**Main Outcomes and Measures:**

*International Classification of Diseases, Ninth Revision* (*ICD-9*) and *International Statistical Classification of Diseases, Tenth Revision, Clinical Modification* (*ICD-10-CM*) codes were used to identify patients with TBI and patients with up to 22 comorbidities within neurological, psychiatric, cardiovascular, and endocrine umbrella groupings. Cox proportional hazard models were used to generate yearly hazard ratios (HRs) from 6 months up to 10 years after a TBI. Models were further stratified by age and ADI score.

**Results:**

The study consisted of 20 400 patients (9264 female [45.4%]; 1576 Black [7.7%], 3944 Latinx [19.3%], and 10 480 White [51.4%]), including 5100 patients with mTBI (median [IQR] age, 36.0 [25.0-51.0] years), 5100 patients with msTBI (median [IQR age, 35.0 [25.0-52.0] years), and 10 200 matched patients in the control group (median [IQR] age, 36.0 [25.0-51.0] years). By ADI score quintile, there were 2757 unexposed patients (27.0%), 1561 patients with mTBI (30.6%), and 1550 patients with msTBI (30.4%) in the lowest (1-2) quintiles and 1523 unexposed patients (14.9%), 769 patients with mTBI (15.1%), and 804 patients with msTBI (15.8%) in the highest quintiles (9-10). TBI of any severity was associated with increased risk of nearly all conditions (mTBI HRs ranged from 1.30; 95% CI, 1.07-1.57 for hypothyroidism to 4.06; 95% CI, 3.06-5.39 for dementia, and msTBI HRs ranged from 1.35; 95% CI, 1.12-1.62 for hypothyroidism to 3.45; 95% CI, 2.73-4.35 for seizure disorder). Separate age and ADI stratifications revealed patient populations at increased risk, including middle-age adults (ages 41-60 years), with increased risk of suicidality (mTBI: HR, 4.84; 95% CI, 3.01-7.78; msTBI: HR, 4.08; 95% CI, 2.51-6.62). Suicidality risk persisted for patients with mTBI in the high ADI subgroup (HR, 2.23; 95% CI, 1.36-3.66).

**Conclusions and Relevance:**

In this cohort study, TBI was a risk factor associated with treatable incident neuropsychiatric and other medical conditions, validating similar findings from a Massachusetts dataset. Additional exploratory findings suggested varying demographic and regional risk patterns, which may generate causal hypotheses for further research and inform clinical surveillance strategies.

## Key Points

**Question:**

Is traumatic brain injury (TBI) associated with long-term risk of incident medical conditions?

**Findings:**

This cohort study compared 10 200 patients with TBI with 10 200 unexposed individuals within a California health care system database and showed longitudinally increased risk of incident post-TBI neuropsychiatric and other medical conditions, validating a recent Massachusetts study. Age and neighborhood features were associated with specific risks, such as 4-fold increased risk of suicidality in middle-aged adults and persisting risk of suicidality among patients with mild TBI affiliated with high neighborhood disadvantage.

**Meaning:**

These findings suggest that understanding regional and neighborhood features in addition to patient-level factors associated with maladaptive clinical phenotypes may optimize post-TBI care.

## Teaser

This cohort study investigates the association of traumatic brain injury with long-term risk of neuropsychiatric and medical conditions.

## Introduction

Traumatic brain injury (TBI) is a major global public health issue affecting 60 million people^1^ annually with adverse effects on quality of life.^2,3,4,5,6,7^ Findings from large observational studies^7,8^ suggest that TBI has long-term clinical consequences, and studies using administrative datasets, patient registries, and survey data have also shown associations between TBI and increased risk of incident chronic conditions in differing patient populations.^9,10,11,12,13,14,15,16,17,18^ Recently, cardiovascular outcomes have been emphasized in part due to treatable risk factors. For example, Izzy et al^11^ used administrative data from a tertiary academic center registry (Boston, Massachusetts) to show associations between TBI and subsequent incidence of multisystem diagnoses, finding increased risk of hypertension in young adults with mild TBI (mTBI). Stewart et al^14^ similarly showed increased risk for cardiovascular disease in mostly young adult, male veterans in the post–September 11 era from the Long-Term Impact of Military-Relevant Brain Injury Consortium-Chronic Effects of Neurotrauma Consortium (LIMBIC-CENC) prospective longitudinal study, while Nyam et al^13^ showed increased risk of post-TBI cardiovascular outcomes via Taiwan’s Longitudinal Health Insurance Database (LHID). Grashow et al^12^ surveyed former American-style football players and found an association between self-reported concussion burden and prevalence of hypertension in this male and largely middle-age population.^12^

Broader characterization of post-TBI sequelae in diverse settings may generate new hypotheses and reveal novel intervention strategies. We therefore sought to validate and extend the totality of findings from Izzy et al^11^ in a large California health care system administrative dataset. Our aim included identification of regional variations in postinjury patterns between the California and Massachusetts datasets. We added a query for menstrual cycle changes and explored the association of neighborhood disadvantage with postinjury outcomes using the area deprivation index (ADI) score,^19^ a composite score of neighborhood features (eg, median income and employment). ADI has only recently been incorporated into TBI outcomes research,^20^ and its clinical associations have not yet been widely studied. We present findings from 5 University of California (UC) health care systems observed over 10 years in 10 200 patients with TBI and 10 200 unexposed individuals, all without preexisting conditions of interest.

## Methods

The University of California, San Francisco, Institutional Review Board waived formal review and informed consent for this cohort study because the study did not meet the definition of human participants research and informed consent would not be possible or indicated. We followed the Strengthening the Reporting of Observational Studies in Epidemiology (STROBE) reporting guideline. We used the UC Health Data Warehouse, which harmonizes electronic health records (EHRs) from 6 sites (Davis, Irvine, Los Angeles, Riverside, San Diego, and San Francisco), with data from more than 8 million patients, 370 million encounters, and 1.1 billion diagnostic codes since 2012.^21^ Harmonization yields the UC Data Discovery Portal, an Observational Medical Outcomes Partnership database unifying fully structured EHR data from multiple health care systems across California.^21^ Some UC sites have younger systems with limited data, and all sites are managed under a single UC-wide initiative.

### Patient Selection and Exposure

Patients with TBI and unexposed individuals were adults aged 18 years or older at the time of injury. Patients with TBI were identified using *International Classification of Diseases, Ninth Revision *(*ICD-9*) codes for TBI (eTable 1 in Supplement 1)^14^ and indexed at the first instance of a TBI diagnostic code in the EHR. Glasgow Coma Scale scores trichotomize injury severity to mild (13-15), moderate (9-12), or severe (3-8) but are not readily coded into EHRs or available in the current dataset. For this reason, we used the Abbreviated Injury Scale for head and neck (0-2 indicates mTBI, and 3-6 indicates moderate to severe TBI [msTBI]) for severity adjudication, a common approach in epidemiologic studies.^22^ Unexposed individuals were indexed on a random medical encounter date. Patients with study comorbidities of interest prior to the index date were excluded. A 10-year time (2013-2022) was used to examine incident conditions from 6 months up to 10 years after the index date. The first 6 months after the index date were excluded to reduce confounding by previously undiagnosed or direct injury–related sequelae.

### Matching

Patients with TBI and unexposed individuals were propensity score–matched on age category, race and ethnicity, sex, site, ADI score, insurance type, and length of time in the respective health care system using Python statistical language version 3.11 (Python Software Foundation) and the PsmPy library version 0.3.13.^23^ Race and ethnicity were self-reported per clinic encounter per institution. Race and ethnicity were reported by the patient via standardized options, including American Indian or Alaska Native, Asian, Black or African American, Latinx, Native Hawaiian or Other Pacific Islander, White, other race or ethnicity, or unknown. If a patient self-identified as Hispanic or Latino and any race, they were categorized as Latinx. Race and ethnicity may be associated with TBI outcomes,^24^ and these data were contained within queried patient records and included in analyses to ensure careful matching and provide descriptive information about the study population. Matching was repeated per UC site given regional demographic distinctions associated with California’s highly diverse population. Patients with TBI and unexposed individuals were required to have at least 1 medical encounter prior to and 6 months after the index date. Matching effect sizes are noted in eFigure 4 in Supplement 1.

### Comorbidities and Age Categories

*ICD-9* and *International Statistical Classification of Diseases, Tenth Revision, Clinical Modification *(*ICD-10-CM*) codes were used to identify comorbidities of interest. We used 4 category groupings (neurological, psychiatric, cardiovascular, and endocrine) and 22 subcategory groupings (21 Massachusetts dataset groupings plus an added grouping for menstrual cycle changes) (eTable 1 in Supplement 1). Age categories were young (aged 18-40 years), middle-age (aged 41-60 years), and older (aged 61-90 years) adults.

### Area Deprivation Index

The ADI score is a composite score of 17 features associated with socioeconomic position within a census block group.^19^ Low and high neighborhood disadvantage are reflected by low and high ADI values, respectively. We focused on lowest (1-2) and highest (9-10) quintiles.

### Statistical Analysis

Cox proportional hazard models generated hazard ratios (HRs) for the 22 comorbidities of interest. Models were adjusted for age, race and ethnicity, sex, ADI score, insurance, and site.

Kaplan-Meier curves were generated for subcategory HRs over time. Separate analyses examined age and ADI stratifications. We used a Bonferroni correction to determine statistical significance (*P* < .002) for multiple tests; all tests were 2-sided. Patients with TBI but no follow-up (excluded in main analyses) were imputed as not acquiring comorbidities of interest for a sensitivity analysis. Data were analyzed using Python statistical language version 3.11 (Python Software Foundation) with PsmPy library version 0.3.13 and Lifelines package version 0.29.0 from August to October 2024.

## Results

### Demographics

**Figure 1.**
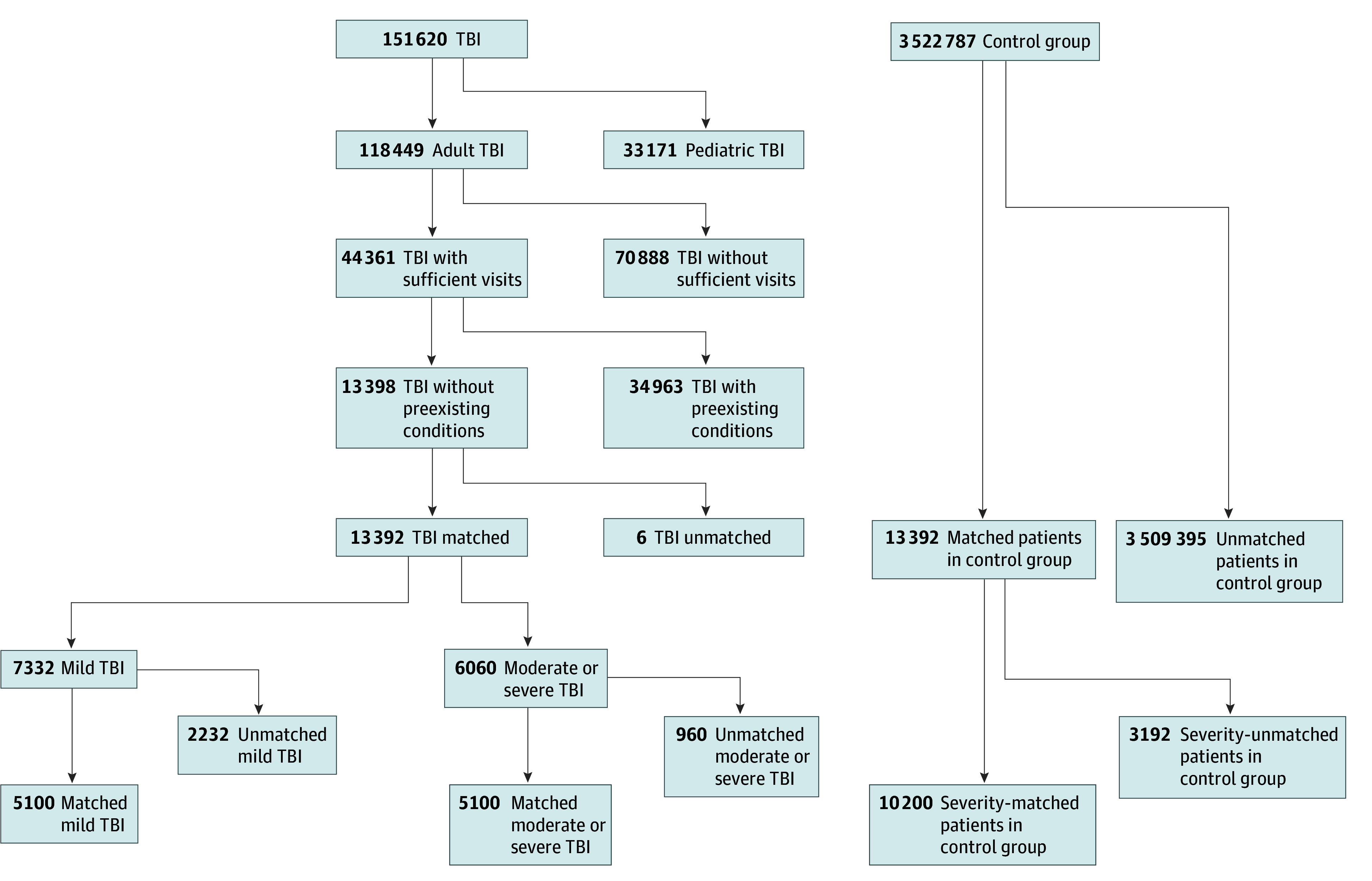
Study Flowchart There were more patients with mild traumatic brain injury (TBI) than those with moderate to severe TBI. Matching was done first for mild TBI and moderate to severe TBI in a 1:1 manner; then, these groups were matched to unexposed patients (control group). Matching occurred after patients with preexisting comorbidities of interest were excluded. A total of 59 223 patients were excluded because they had no prior encounters, 61 116 patients because they had no follow-up, and 63 103 because they had no prior nor follow-up encounters.

We identified 118 449 adult patients with TBI. After applying inclusion and exclusion criteria, we analyzed 20 400 patients (9264 female [45.4%]; 1576 Black [7.7%], 3944 Latinx [19.3%], and 10 480 White [51.4%]), including 5100 patients with mTBI (median [IQR] age, 36.0 [25.0-51.0] years), 5100 patients with msTBI (median [IQR] age, 35.0 [25.0-52.0] years), and 10 200 matched unexposed individuals (median [IQR] age, 36.0 [25.0-51.0] years) (Figure 1; Table 1). By ADI score quintile, there were 2757 unexposed patients (27.0%), 1561 patients with mTBI (30.6%), and 1550 patients with msTBI (30.4%) in the lowest (1-2) quintiles and 1523 unexposed patients (14.9%), 769 patients with mTBI (15.1%), and 804 patients with msTBI (15.8%) in the highest quintiles (9-10).

**Table 1.** Baseline Patient Characteristics

Characteristic	Patients, No. (%)		
	Control group (n = 10 200)	mTBI (n = 5100)	msTBI (n = 5100)
Age, median (IQR), y	36.0 (25.0-51.0)	36.0 (25.0-51.0)	35.0 (25.0-52.0)
Race and ethnicity			
American Indian or Alaska Native	10 (<0.1)	5 (<0.1)	5 (<0.1)
Asian	906 (8.9)	453 (8.9)	453 (8.9)
Black or African American	788 (7.7)	394 (7.7)	394 (7.7)
Latinx	1972 (19.3)	986 (19.3)	986 (19.3)
Native Hawaiian or Other Pacific Islander	26 (0.3)	13 (0.3)	13 (0.3)
White	5240 (51.4)	2620 (51.4)	2620 (51.4)
Other race	544 (5.3)	272 (5.3)	272 (5.3)
Unknown	714 (7.0)	357 (7.0)	357 (7.0)
Sex			
Female	4632 (45.4)	2316 (45.4)	2316 (45.4)
Male	5568 (54.6)	2784 (54.6)	2784 (54.6)
Site			
UCD	3022 (29.6)	1511 (29.6)	1511 (29.6)
UCI	1166 (11.4)	583 (11.4)	583 (11.4)
UCLA	3184 (31.2)	1592 (31.2)	1592 (31.2)
UCSD	1842 (18.1)	921 (18.1)	921 (18.1)
UCSF	986 (9.7)	493 (9.7)	493 (9.7)
Health insurance coverage			
NA	386 (3.8)	149 (2.9)	237 (4.6)
Medicare Medi-Cal	275 (2.7)	129 (2.5)	146 (2.9)
Medicaid	3214 (31.5)	1501 (29.4)	1713 (33.6)
Medicare	341 (3.3)	183 (3.6)	158 (3.1)
Medicare Advantage	144 (1.4)	53 (1.0)	91 (1.8)
Private	5689 (55.8)	3006 (58.9)	2683 (52.6)
Veteran	151 (1.5)	79 (1.5)	72 (1.4)
ADI score			
1	1612 (15.8)	972 (19.1)	955 (18.7)
2	1145 (11.2)	589 (11.5)	595 (11.7)
3	1077 (10.6)	518 (10.2)	451 (8.8)
4	1105 (10.8)	495 (9.7)	516 (10.1)
5	945 (9.3)	507 (9.9)	433 (8.5)
6	1035 (10.1)	421 (8.3)	454 (8.9)
7	872 (8.5)	422 (8.3)	436 (8.5)
8	886 (8.7)	407 (8.0)	456 (8.9)
9	878 (8.6)	411 (8.1)	428 (8.4)
10	645 (6.3)	358 (7.0)	376 (7.4)
No. encounters after index			
Median (IQR)	9.0 (4.0-21.0)	15.0 (7.0-38.0)	15.0 (6.0-39.0)
Mean (SD)	20.65 (37.83)	36.28 (67.41)	37.30 (72.78)
No. encounters 1 y after index			
Median (IQR)	3.0 (1.0-8.0)	5.0 (2.0-14.0)	5.0 (1.0-13.0)
Mean (SD)	6.64 (11.80)	10.63 (15.94)	10.87 (18.52)

Abbreviations: ADI, area deprivation index; mTBI, mild traumatic brain injury; msTBI, moderate to severe traumatic brain injury; NA, not applicable; UCD, University of California, Davis; UCI, University of California, Irvine; UCLA, University of California, Los Angeles; UCSD, University of California, San Diego; UCSF, University of California, San Francisco.

### Follow-Up Period Distribution

Beyond the first 6 months after the index date, there was a median [IQR] of 3.4 (1.6-6.0) years of follow-up for patients with mTBI and 3.6 (1.6-6.6) years of follow-up for patients with msTBI. Among unexposed individuals, this median (IQR) for follow-up was 3.5 (1.6-6.2) years.

### Comorbidities

**Figure 2.**
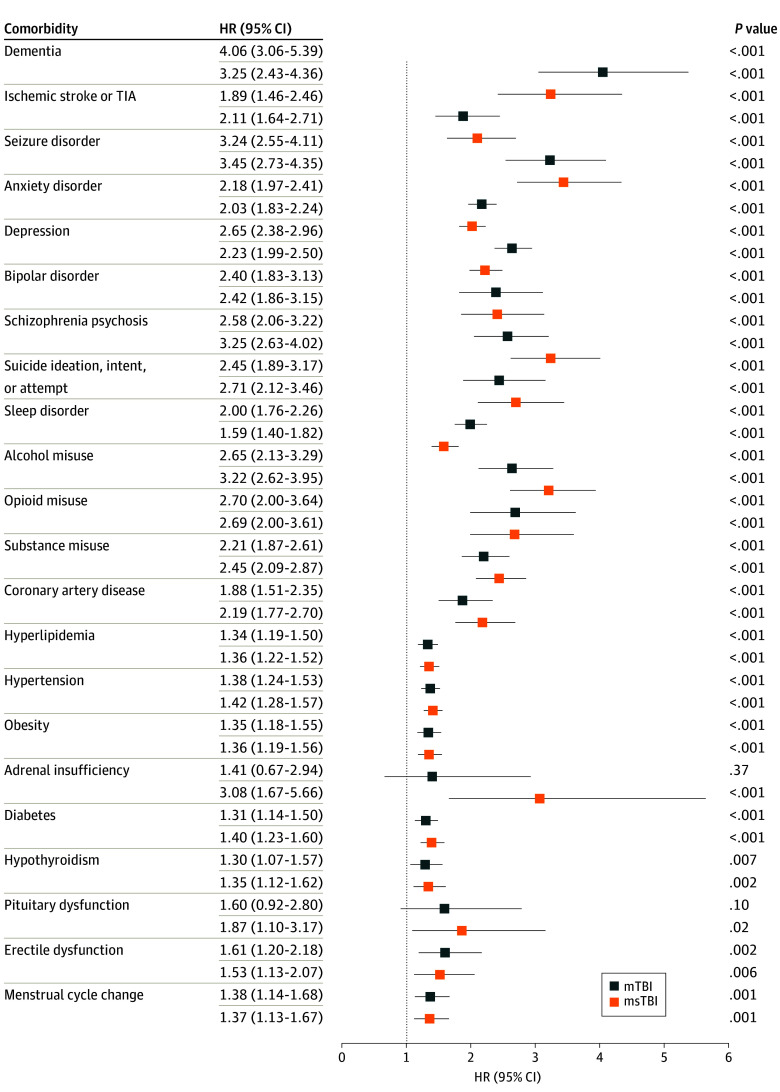
Risk of Comorbidities, Original Model HR indicates hazard ratio; mTBI, mild traumatic brain injury; msTBI, moderate to severe traumatic brain injury; TIA, transient ischemic attack; whiskers, 95% CIs.

**Figure 3.**
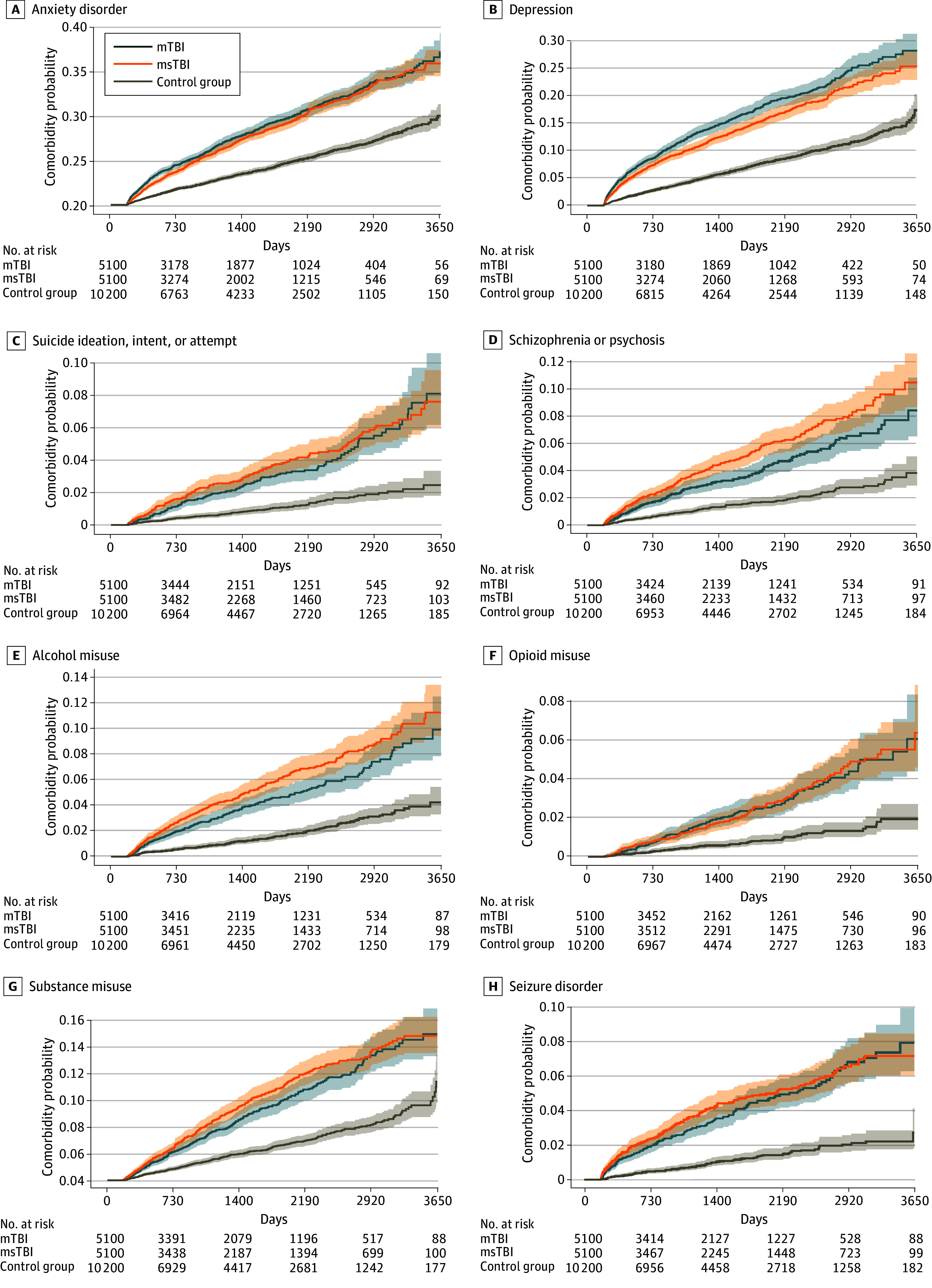
Select Kaplan-Meier Curves Longitudinal risk of select incident conditions is presented. mTBI indicates mild traumatic brain injury; msTBI, moderate to severe traumatic brain injury.

Compared with being unexposed, TBI was associated with increased risk of all conditions in neurological, psychiatric, and cardiovascular categories and several conditions in the endocrine category (Table 2; Figure 2). Select Kaplan-Meier subcategory curves are shown in Figure 3 (all curves are shown in eFigure 1 in Supplement 1). Age-stratified HRs are shown in eTable 2 and eFigure 2 in Supplement 1. ADI quintile–stratified HRs are shown in eTable 3 and eFigure 3 in Supplement 1. Sensitivity analysis (TBI but no follow-up imputed as no incident comorbidities) revealed similar results with slightly lower HRs and with persisting significance for most outcomes. Notable findings are highlighted subsequently. All HRs represent patients with TBI vs unexposed individuals.

**Table 2.** TBI and Risk of Comorbidities, Original Model^a^

Comorbidity	Unexposed group		mTBI				msTBI			
	Patients, No.	Follow-up, person-days	Patients, No.	Follow-up, person-days	HR (95% CI)	*P* value	Patients, No.	Follow-up, person-days	HR (95% CI)	*P* value
Neurological outcomes										
Ischemic stroke or TIA	135	159 526	107	139 949	1.89 (1.46-2.46)	<.001	129	157 409	2.11 (1.64-2.71)	<.001
Seizure disorder	102	125 443	171	167 893	3.24 (2.55-4.11)	<.001	197	172 814	3.45 (2.73-4.35)	<.001
Dementia	86	102 625	140	156 142	4.06 (3.06-5.39)	<.001	132	119 669	3.25 (2.43-4.36)	<.001
Psychiatric outcomes										
Depression	603	665 890	700	609 154	2.65 (2.38-2.96)	<.001	632	649 419	2.23 (1.99-2.50)	<.001
Bipolar disorder	104	128 077	114	129 462	2.40 (1.83-3.13)	<.001	120	132 876	2.42 (1.86-3.15)	<.001
Schizophrenia or psychosis	138	168 389	164	179 356	2.58 (2.06-3.22)	<.001	230	249 611	3.25 (2.63-4.02)	<.001
Anxiety disorder	739	896 503	752	727 737	2.18 (1.97-2.41)	<.001	738	794 425	2.03 (1.83-2.24)	<.001
Sleep disorder	550	640 139	481	514 135	2.00 (1.76-2.26)	<.001	417	491 357	1.59 (1.40-1.82)	<.001
Suicidality or attempt	91	146 268	129	154 328	2.45 (1.89-3.17)	<.001	158	168 425	2.71 (2.12-3.46)	<.001
Substance misuse	267	359 378	280	327 033	2.21 (1.87-2.61)	<.001	336	363 640	2.45 (2.09-2.87)	<.001
Opioid misuse	69	97 994	100	125 340	2.70 (2.00-3.64)	<.001	110	141 944	2.69 (2.00-3.61)	<.001
Alcohol misuse	145	189 617	187	198 271	2.65 (2.13-3.29)	<.001	252	260 284	3.22 (2.62-3.95)	<.001
Cardiovascular outcomes										
Hypertension	841	1 055 213	535	608 665	1.38 (1.24-1.53)	<.001	608	674 231	1.42 (1.28-1.57)	<.001
Hyperlipidemia	789	980 771	484	564 413	1.34 (1.19-1.50)	<.001	514	630 838	1.36 (1.22-1.52)	<.001
Obesity	581	683 910	354	467 705	1.35 (1.18-1.55)	<.001	357	452 480	1.36 (1.19-1.56)	<.001
Coronary artery disease	219	232 404	148	196 953	1.88 (1.51-2.35)	<.001	180	257 929	2.19 (1.77-2.70)	<.001
Endocrine outcomes										
Hypothyroidism	249	311 487	178	190 126	1.30 (1.07-1.57)	.007	180	215 909	1.35 (1.12-1.62)	.002
Pituitary dysfunction	28	31 510	21	21 308	1.60 (0.92-2.80)	.10	25	28 806	1.87 (1.10-3.17)	.02
Diabetes	554	705 039	335	412 096	1.31 (1.14-1.50)	<.001	383	501 103	1.40 (1.23-1.60)	<.001
Adrenal insufficiency	29	22 458	14	12 733	1.41 (0.67-2.94)	.37	26	31 209	3.08 (1.67-5.66)	<.001
Erectile dysfunction	102	111 569	77	101 876	1.61 (1.20-2.18)	.002	79	102 048	1.53 (1.13-2.07	.006
Menstrual cycle change	235	259 423	173	178 652	1.38 (1.14-1.68)	<.001	175	198 220	1.37 (1.13-1.67)	<.001

Abbreviations: HR, hazard ratio; mTBI, mild traumatic brain injury; msTBI, moderate to severe traumatic brain injury; TIA, transient ischemic attack.

^a^ Outcomes are adjusted for age, race and ethnicity, and sex. Cox proportional hazard results per condition are presented, plus the number of patients and person-days of follow-up. Subcategory groupings included dementia, seizure, and TIA or stroke (neurological outcomes); depression, bipolar disorder, schizophrenia or psychosis, anxiety disorder, sleep disorder, suicidality or suicide attempt, substance misuse, opioid misuse, and alcohol misuse (psychiatric outcomes); hypertension, hyperlipidemia, obesity, and coronary artery disease (cardiovascular outcomes); and hypothyroidism, pituitary dysfunction, diabetes, adrenal insufficiency, erectile dysfunction, and menstrual cycle changes (endocrine outcomes).

#### Neurological Disorders

There was an increased risk of incident seizure disorder for patients with TBI vs unexposed patients (mTBI: HR, 3.24; 95% CI, 2.55-4.11; msTBI: HR, 3.45; 95% CI, 2.73-4.35), and injury severity conferred a dose response (eTable 2 in Supplement 1). There was also an increased risk of incident dementia among patients with TBI (mTBI: HR, 4.06; 95% CI, 3.06-5.39; msTBI: HR, 3.25; 95% CI, 2.43-4.36) (Table 2; Figure 2).

##### Age stratification

Increased seizure and dementia risk persisted across all ages for patients with TBI vs unexposed patients. Among patients with mTBI, seizure HRs ranged from 2.43 (95% CI, 1.77-3.34) for ages 18 to 40 years to 5.13 (95% CI, 2.28-11.55) for ages 61 to 90 years, while among patients with msTBI, seizure HRs ranged from 2.80 (95% CI, 2.06-3.79) for ages 18 to 40 years to 6.83 (95% CI, 3.15-14.84) for ages 61 to 90 years. Among patients with mTBI, dementia HRs ranged from 3.50 (95% CI, 2.46-4.98) for ages 61 to 90 years to 10.27 (95% CI, 4.08-25.87) for ages 18 to 40 years, while among patients with msTBI, dementia HRs ranged from 2.58 (95% CI, 1.42-4.71) for ages 41 to 60 years to 7.67 (95% CI, 2.99-19.68) for ages 18 to 40 years. Stroke and transient ischemic attack risk was significantly increased in older adults (ages 61-90 years) with TBI only (mTBI: HR, 2.22; 95% CI, 1.51-3.27; msTBI: HR, 2.68; 95% CI, 1.85-3.87) (eTable 2 and eFigure 2 in Supplement 1).

##### ADI stratification

Patients in the lowest (least disadvantaged; ADI, 1-2; HR range, 2.53; 95% CI, 1.48-4.34 for patients with mTBI to 2.55; 95% CI, 1.50-4.33 for patients with msTBI) and highest (most disadvantaged; ADI, 9-10; HR range, 2.36; 95% CI, 1.38-4.05 for patients with mTBI to 3.16; 95% CI 1.91-5.24 for patients with msTBI) quintiles had increased risk of seizure disorder after any TBI. Any TBI was associated with increased risk of dementia in the lowest ADI quintile (msTBI: HR, 2.75; 95% CI, 1.57-4.81; mTBI: HR, 5.04; 95% CI, 3.01-8.45), and msTBI was associated with increased risk of dementia in the highest ADI quintile (HR, 6.77; 95% CI, 2.98-15.37). Patients in the lowest but not highest ADI quintile had increased risk of transient ischemic attack or stroke after msTBI (HR, 3.02; 95% CI, 1.86-4.89) (eTable 3 and eFigure 3 in Supplement 1).

#### Psychiatric Disorders

For most subcategories, there was at least a 2-fold increase in risk of incident psychiatric conditions after any TBI compared with no exposure. Among patients with mTBI, HRs ranged from 2.00 (95% CI, 1.76-2.26) for sleep disorder to 2.70 (95% CI, 2.00-3.64) for opioid misuse, while among patients with msTBI, HRs ranged from 1.59 (95% CI, 1.40-1.82) for sleep disorder to 3.25 (95% CI, 2.63-4.02) for schizophrenia or psychosis (Table 2; Figure 2).

##### Age stratification

TBI compared with no exposure was associated with increased risk of all psychiatric subcategories in young and middle-aged adults, including increased risk of suicidality in middle-aged adults (mTBI: HR, 4.84; 95% CI, 3.01-7.78; msTBI: HR, 4.08; 95% CI, 2.51-6.62). Older adults with TBI remained at increased risk of incident alcohol misuse and mood, sleep, and psychotic disorders; for example, psychosis or schizophrenia HRs were 3.63 (95% CI, 1.94-6.79) for msTBI and 4.82 (95% CI, 2.64-8.81) for mTBI (eTable 2 and eFigure 2 in Supplement 1).

##### ADI stratification

There was at least a 3-fold increased risk of alcohol misuse and 2-fold increased risk of depression and substance misuse across low and high quintiles with TBI vs no exposure. Patients with any TBI in the highest but not lowest ADI quintile were at increased risk of opioid misuse (mTBI: HR, 3.54; 95% CI, 1.88-6.68; msTBI: HR, 3.81; 95% CI, 2.04-7.11). Patients with any TBI in the lowest but not highest ADI quintile were at increased risk of sleep disorders (mTBI: HR, 2.13; 95% CI, 1.71-2.65; msTBI: HR, 1.56; 95% CI, 1.24-1.97). Patients with mTBI in the highest but not lowest ADI quintile were uniquely at increased risk of bipolar disorder (HR, 2.73; 95% CI, 1.50-4.98) and suicidality (HR, 2.23; 95% CI, 1.36-3.66) compared with unexposed individuals (eTable 3 and eFigure 3 in Supplement 1).

#### Cardiovascular Disease

Risk of incident cardiovascular conditions after any TBI was uniformly increased. HRs ranged from 1.34 (95% CI, 1.19-1.50) for hyperlipidemia among patients with mTBI to 2.19 (95% CI, 1.77-2.70) for coronary artery disease (CAD) among patients with msTBI (Table 2; Figure 2).

##### Age stratification

There were no significant findings for cardiovascular outcomes among young adults with TBI. Middle-aged adults with msTBI had increased risk of CAD and hypertension. Older adults with any TBI had increased risk of all subcategories of cardiovascular disease. Among patients with mTBI, HRs ranged from 1.70 (95% CI, 1.40-2.08) for hyperlipidemia to 2.67 (95% CI, 1.90-3.75) for obesity, while among patients with msTBI, HRs ranged from 1.67 (95% CI, 1.40-1.98) for hypertension to 2.61 (95% CI, 1.92-3.54) for CAD (eTable 2 and eFigure 2 in Supplement 1).

##### ADI stratification

Among patients in the lowest ADI quintile, any TBI vs no exposure was associated with increased risk of hyperlipidemia (mTBI: HR, 1.62; 95% CI, 1.34-1.98; msTBI: HR, 1.68; 95% CI, 1.39-2.03), hypertension (mTBI: HR, 1.83; 95% CI, 1.48-2.27; msTBI: 1.61; 95% CI, 1.30-1.99), and CAD (mTBI: HR, 2.55; 95% CI, 1.65-3.94; msTBI: HR, 2.39; 95% CI, 1.55-3.66) but not obesity. Among patients in the highest ADI quintile, msTBI was associated with increased risk of CAD compared with no exposure (HR, 2.39; 95% CI, 1.51-3.77) but no other conditions (eTable 3 and eFigure 3 in Supplement 1).

#### Endocrine Disorders

Most subcategories demonstrated an association between TBI compared with no exposure and increased risk for target conditions, including added codes for menstrual cycle changes. There were mixed outcomes for injury severity. There was an association between any TBI and increased risk of diabetes (mTBI: HR, 1.31; 95% CI, 1.14-1.50; msTBI: 1.40; 95% CI, 1.23-1.60) and hypothyroidism (mTBI: HR, 1.30; 95% CI, 1.07-1.57; msTBI: HR, 1.35; 95% CI, 1.12-1.62); msTBI was associated with increased risk of adrenal insufficiency (HR, 3.08; 95% CI, 1.67-5.66).

##### Age stratification

Among young adults, any TBI was associated with increased risk of erectile dysfunction (mTBI: HR, 2.59; 95% CI, 1.54-4.36; msTBI: HR, 3.30; 95% CI, 1.93-5.67) and mTBI was associated with increased risk of menstrual cycle changes (HR, 1.44; 95% CI, 1.16-1.80) compared with no exposure. Middle-aged patients with msTBI had increased risk of incident pituitary dysfunction compared with patients without exposure (HR, 6.09; 95% CI, 2.43-15.24) and no other condition. Among older adults, TBI was associated with increased risk for diabetes compared with no exposure (mTBI: HR, 1.74; 95% CI, 1.36-2.22; msTBI: HR, 1.90; 95% CI, 1.50-2.41) and hypothyroidism (mTBI: HR, 1.81; 95% CI, 1.33-2.46; msTBI: HR, 1.64; 95% CI, 1.20-2.23) and no other condition (eTable 2 and eFigure 2 in Supplement 1).

##### ADI stratification

Among patients in the lowest ADI quintile, any TBI was associated with increased risk for diabetes compared with no exposure (mTBI: HR, 1.60; 95% CI, 1.24-2.07; msTBI: HR, 1.61; 95% CI, 1.26-2.06) and no other condition. There were no significant findings in the highest ADI quintile (eTable 3 and eFigure 3 in Supplement 1).

## Discussion

In this cohort study of a large California health care system administrative dataset, 10 200 patients with history of TBI had longitudinally increased risk of chronic conditions compared with unexposed individuals. Incident neuropsychiatric conditions occurred among all age groups, with unacceptably high risk of many mental health disorders, including alcohol and substance misuse and suicidality. TBI neuropathophysiology comprises a convergence of mechanism (eg, blast vs single vs repetitive or subconcussive injuries),^25,26^ inflammatory and microvascular changes,^27,28,29,30^ structural and functional network injury, and individual characteristics (eg, age, genetics, and comorbidities).^29^ Postinjury neuropsychiatric manifestations represent behavioral end points that mimic primary neuropsychiatric disorders but with an evolving neuropathophysiology that differs from noninjury substrates.^6,7,29,31,32,33^ A paucity of TBI-specific diagnostic codes compels specialty and nonspecialty clinicians to use approximate diagnoses during a health care encounter.^31^ Despite limitations inherent to administrative data and specifically related to TBI, our findings confirm that TBI was a risk factor associated with diverse clinical outcomes, validating Izzy et al,^11^ complementing prior epidemiologic studies, and supporting the generalizability of findings.^10,11,12,13,14,15,16,17,18^ Individual, neighborhood, population, regional, and systemic circumstances are deserving of further study to personalize postinjury care.^7^ Salient findings are discussed subsequently.

### Area Deprivation Features

Socioeconomic status elements, such as insurance coverage and type, are associated with post-TBI hospital length of stay or access to rehabilitation services and thus potentially recovery patterns.^24,34^ Prospectively collected data have also shown the association of ADI (which does not account for insurance features) with persisting symptoms 6 months after mTBI.^20^ We leveraged our large dataset and stringent matching, including on insurance features, to examine ADI extremes. We found increased incidence of most neuropsychiatric conditions in low and high ADI quintiles, suggestive of some degree of indiscriminate or intrinsic^29,32,35^ neuropsychiatric outcomes associated with TBI. However, despite a smaller cohort size, high ADI was uniquely associated with incident bipolar disorder, opioid misuse, and suicidality diagnoses, particularly among patients with mTBI; these results require careful study to establish directional or causal relationships and to amplify outreach efforts against systemic barriers to care. In contrast, low ADI was associated with more incident cardiovascular conditions, which may reflect bias due to the size of this ADI subgroup or differences in EHR documentation due to consistent access to care.

### Neurological Outcomes

The California dataset identified increased risk of post-TBI dementia diagnoses across all age groups, in contrast to the Massachusetts dataset. Association of TBI with downstream neurodegenerative diagnoses in middle or older age adults^17,18^ is robustly described, but study of young adults compared with carefully selected unexposed cohorts is needed.^36^ A cross-sectional LIMBIC-CENC prospective longitudinal study among military service members and veterans (mean age, 39.7 years) did not find evidence of persisting cognitive impairment among relatively young individuals with a history of single or repetitive mTBI.^37^ In the California dataset, large HRs and wide CIs for dementia diagnoses among young adults may reflect previously mentioned coding considerations, rare diagnostic events, or both. Findings across age groups require scrutiny to establish a symptom time course and determine how synergistic comorbidities, such as mood, neuroendocrine, or sleep impairment, may modify resilience or risk factors against neurodegenerative disease (eg, older age at the time of injury).^17,18,29,38^

### Psychiatric Outcomes

Post-TBI psychiatric conditions are mechanistically understudied and underrecognized.^31,39^ Young and middle-aged adults in the California dataset were at risk of all conditions queried. Depression and alcohol misuse persisted across all age group stratifications and in low and high ADI quintiles. California findings may in part reflect the impact of the global COVID-19 pandemic (the California study period was 2013-2022 vs the Massachusetts study period ending in 2015) or the regional mental health crisis.^40^ However, our findings, including that of increased risk of suicidality, are similar to those of other studies with differing study designs or patient populations, including civilian and military cohorts.^11,41,42,43^ In addition to distinct neuropathophysiology^31,32^ subserving clinical phenotypes, impaired frontotemporal-subcortical-thalamic circuitry and related behaviors (eg, impulsivity, emotional dysregulation, executive dysfunction, and reward-seeking) yield reciprocal and overlapping risks for symptom chronicity, recurrent TBI, and other chronic conditions even in mTBI. Injury context (eg, sport, war, assault, or accident) and prior injury history are important modifiers of an individual’s neurophysiologic or endocrine response and therefore symptom burden.^7^ We join other groups in emphasizing the critically urgent and unmet need for enhanced mental health care for patients with a history of TBI. Understanding mechanism, directionality, and interaction of exacerbated or de novo conditions will facilitate proper screening and intervention strategies.^44,45^

### Endocrine Outcomes

There is a lack of comprehensive longitudinal data regarding incidence and timing of neuroendocrine dysfunction after TBI. Neuroendocrine dysfunction has mechanistically been associated with more severe injuries and is potentially underrecognized otherwise.^46,47^ In the California dataset, there was mixed association of TBI with endocrine disorders. Yang et al^48^ used the Taiwanese LHID to study select post-TBI endocrinopathies in patients without target conditions and found a 2-fold increased risk of pituitary disorders 1 year after TBI compared with no exposure, with widening cumulative incidence curves between patients with TBI and unexposed patients over 5 years. We used different *ICD* codes to study a broader range of endocrine conditions, but dedicated longitudinal research is needed to guide clinical screening strategies like type and timing of laboratory evaluation of neuroendocrine dysfunction; this should complement routine clinical review of systems querying sexual dysfunction, menstrual cycle changes, and mood or cognitive symptoms to identify treatable or reversible conditions. It is possible that earlier screening may identify a counterproductive neuroendocrine milieu that may be another clinical intervention target.

### Cardiovascular Outcomes

For cardiovascular conditions, our findings align with the Massachusetts dataset and other studies described previously,^12,13,14^ along with a prior study of a California emergency department adult cohort matched to a control group with non-TBI trauma.^15^ In contrast to the Massachusetts findings, age stratification did not identify increased cardiovascular risk in young adults. These differences among the California, Massachusetts, and other datasets may in part be attributed to demographic variations. The California dataset population had a 10-year younger median age and was more racially and ethnically diverse compared with the Massachusetts dataset. These differences align with known census-level data. California is the most populous US state, with median age of 37.9 years, compared with a Massachusetts median age of 40.3 years, and West Coast risk for cardiovascular disease in adults is lower compared with that of the Northeast.^49,50,51,52,53^

### Limitations

Our study has limitations. Administrative data rely on exposure to the patient populations of interest, selection of appropriate diagnostic codes during a health care encounter (most of which are developed for non-TBI use cases), and availability of desired data elements. Glasgow Coma Scale scores for severity adjudication are often not available. Collectively, these coding hygiene features may yield selection biases. We intentionally used the methods of Izzy et al^11^ to draw direct comparisons between the California and Massachusetts datasets; this restricted our study population and eliminated patients with preexisting conditions of interest. The studied conditions often interact with each other, pose risk of TBI, or are associated with recovery trajectories,^7,54^ potentially yielding collider bias. We did not include a non-TBI trauma cohort to examine trauma-specific risk contributions for comorbidities of interest. We did not stratify by sex, although our cohorts were 45% female. We could not account for injury context, mechanism, or recurrence. Additionally, there was a clear signal for differing associations of neighborhood features, but the studied patient population had a smaller proportion of patients within the high vs low ADI quintile.

## Conclusions

In this cohort study of 10 200 patients with TBI, we found increased risk of treatable incident neuropsychiatric and other systemic conditions, adding to a growing body of literature reframing TBI as a chronic condition. At minimum, cross-sectional and longitudinal clinical screening and treatment strategies should target mental and cognitive health, with vigilance regarding suicidality and with consideration of cross-domain conditions, including sleep disturbance, substance misuse (especially of alcohol), vascular disease, and endocrine dysfunction. Targeted causal investigation is needed to inform optimal outreach, surveillance, testing, and treatment given that many of the 22 subcategories queried have multidirectional associations and variable patterns across demographic and neighborhood features.

## Notes

Supplement 1. **eTable 1.** *ICD-9* and *ICD-10* Codes Used **eFigure 1.** Kaplan-Meier Curves **eTable 2.** Cox Proportional Hazard Ratios Stratified by Age **eFigure 2.** Cox Proportional Hazard Ratios Stratified by Age, Whisker Plots **eTable 3.** Cox Proportional Hazards Model Stratified by Low and High ADI Quintiles **eFigure 3.** Cox Proportional Hazards Model Stratified by Low and High ADI Quintiles, Whisker Plots **eFigure 4.** Post Matching Effect Sizes

Supplement 2.
Data Sharing Statement
