# Supplementary material for: Traumatic Brain Injury and Risk of Incident Comorbidities
Source: JAMA Netw Open. 2024 Dec 12;7(12):e2450499. doi: 10.1001/jamanetworkopen.2024.50499 (PMC11638795; doi:10.1001/jamanetworkopen.2024.50499)
Supplement: Supplement 1. — eTable 1. ICD-9 and ICD-10 Codes Used eFigure 1. Kaplan-Meier Curves eTable 2. Cox Proportional Hazard Ratios Stratified by Age eFigure 2. Cox Proportional Hazard Ratios Stratified by Age, Whisker Plots eTable 3. Cox Proportional Hazards Model Stratified by Low and High ADI Quintiles eFigure 3. Cox Proportional Hazards Model Stratified by Low and High ADI Quintiles, Whisker Plots eFigure 4. Post Matching Effect Sizes [file jamanetwopen-e2450499-s001.pdf]

Supplemental Online Content

Halabi C, Izzy S, DiGiorgio AM, et al. Traumatic brain injury and risk of incident comorbidities. *JAMA Netw Open*. 2024;7(12):e2450499. doi:10.1001/jamanetworkopen.2024.50499

eTable 1. *ICD-9* and *ICD-10* Codes Used

eFigure 1. Kaplan-Meier Curves

eTable 2. Cox Proportional Hazard Ratios Stratified by Age

eFigure 2. Cox Proportional Hazard Ratios Stratified by Age, Whisker Plots

eTable 3. Cox Proportional Hazards Model Stratified by Low and High ADI Quintiles

eFigure 3. Cox Proportional Hazards Model Stratified by Low and High ADI Quintiles, Whisker Plots

eFigure 4. Post Matching Effect Sizes

This supplemental material has been provided by the authors to give readers additional information about their work.

© 2024 Halabi C et al. *JAMA Network Open.*

Disorder	ICD-9	ICD-10
**Cardiovascular Disorders**		
Hypertension	401, 402, 403, 404, 405	I10, I11, I12, I13, I15, I16
Hyperlipidemia	272	E78
Obesity	278	E66, Z68.25-Z68.44
Coronary artery disease	410, 411, 412, 413, 414	I21, I22, I23, I24, I25
**Endocrine disorders**		
Hypothyroidism	244	E03
Pituitary dysfunction	253	E23
Diabetes mellitus	250, 790.29, 790.21	E08, E09, E11, E13
Adrenal insufficiency	255.4-255.9	E27.0-E27.7
Erectile dysfunction	607.84	N52, F52.21
Menstral Cycle Changes	626	N92
**Psychiatric disorders**		
Depression	296.2, 296.3, 296.8, 300.4, 311	F32, F33
Bipolar disorder	296.4, 296.5, 296.6, 296.7	F25, F31
Schizophrenia/ psychosis	295, 296.89, 296.9, 298	F06, F20, F22, F23, F28, F29
Anxiety disorder	300, 300.2, 308, 309	F41, F40.8, F40.9, F43.2
Sleep disorder	307.4, 327, 780.5, 347	F51, G47
Suicide ideation/intent/attempt	E950 - E958, V62.84	R45. 851, X83, T40, Z91.5
Substance misuse	304.1-304.6, 304.8-304.9, 305.2, 305.3, 305.4, 305.6-305.9	F12, F13, F14, F15, F16, F18, F19
Opioid misuse	304.0, 304.7, 305.5	F11
Alcohol misuse	303, 305.0	F10
**Neurological disorders**		
Ischemic stroke/TIA	433, 434, 435, 436, 437	I63, I65, I66, I67
Dementia	290.0-290.4, 290.8, 290.9, 294.1, 294.2, 331	F01, F02, F03, G30, G31
Seizure disorder	780.33, 780.39, 345	R56.1, R56.9, G40

eTable 1, ICD-9 and ICD-10 codes used.

© 2024 Halabi C et al. *JAMA Network Open.*

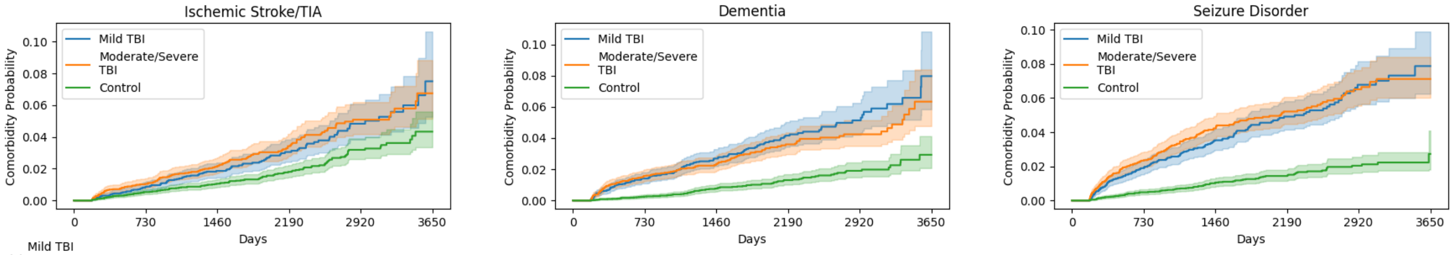

Ischemic Stroke/TIA

Mild TBI						
At risk	5100	3447	2157	1243	536	81
Censored	0	1617	2878	3772	4463	4912
Events	0	36	65	85	101	107
Moderate/Severe						
TBI		3503	2279	1464	727	100
At risk	5100	1550	2741	3532	4249	4871
Censored	0	47	80	104	124	129
Events	0					
Control		6955	4449	2705	1244	179
At risk	10200	3200	5676	7394	8828	9886
Censored	0	45	75	101	128	135
Events	0					

Dementia

Mild TBI						
At risk	5100	3431	2146	1245	540	88
Censored	0	1607	2856	3732	4428	4872
Events	0	62	98	123	132	140
Moderate/Severe						
TBI		3489	2285	1476	744	101
At risk	5100	1541	2723	3507	4231	4867
Censored	0	70	92	117	125	132
Events	0					
Control		6974	4468	2720	1256	182
At risk	10200	3205	5683	7412	8864	9932
Censored	0	21	49	68	80	86
Events	0					

Seizure Disorder

Mild TBI						
At risk	5100	3414	2127	1227	528	88
Censored	0	1603	2844	3720	4404	4841
Events	0	83	129	153	168	171
Moderate/Severe						
TBI		3467	2245	1448	723	99
At risk	5100	1529	2695	3473	4184	4804
Censored	0	104	160	179	193	197
Events	0					
Control		6956	4458	2718	1258	182
At risk	10200	3203	5672	7396	8843	9916
Censored	0	41	70	86	99	102
Events	0					

eFigure 1a, Kaplan-Meier Curves, Neurological Disorders.

© 2024 Halabi C et al. *JAMA Network Open.*

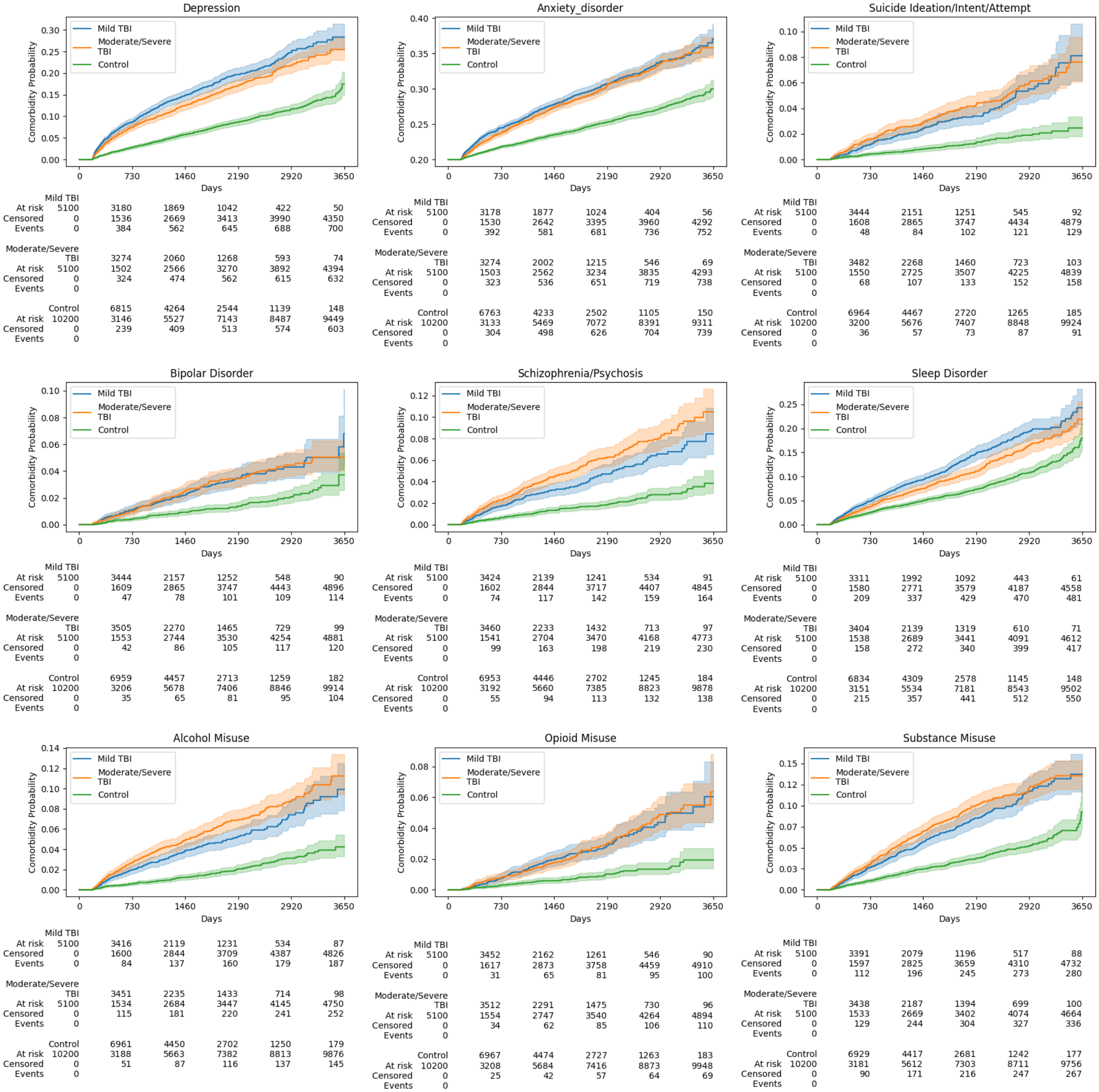

eFigure 1b, Kaplan-Meier Curves, Psychiatric Disorders.

© 2024 Halabi C et al. *JAMA Network Open.*

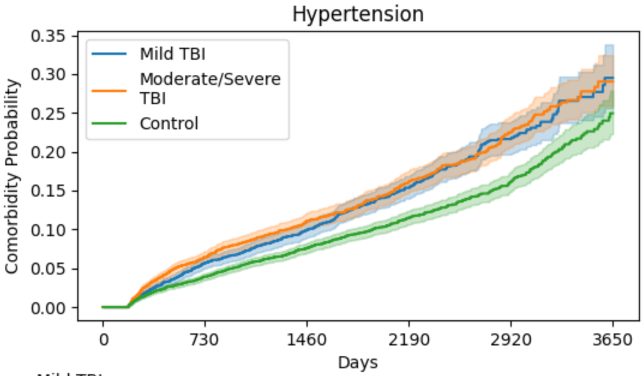

Mild TBI						
At risk	5100	3287	1977	1077	434	65
Censored	0	1571	2763	3569	4153	4500
Events	0	242	360	454	513	535
Moderate/Severe						
TBI		3314	2077	1276	589	69
At risk	5100	1503	2604	3313	3931	4423
Censored	0	283	419	511	580	608
Events	0					
Control		6748	4187	2475	1087	142
At risk	10200	3111	5461	7033	8331	9217
Censored	0	341	552	692	782	841
Events	0					

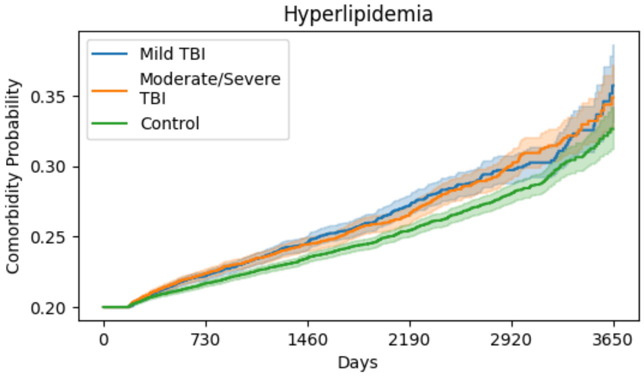

Mild TBI						
At risk	5100	3325	1989	1077	436	56
Censored	0	1585	2789	3611	4204	4560
Events	0	190	322	412	460	484
Moderate/Severe						
TBI		3362	2106	1292	578	66
At risk	5100	1531	2667	3404	4039	4520
Censored	0	207	327	404	483	514
Events	0					
Control		6768	4208	2479	1063	132
At risk	10200	3145	5501	7090	8402	9279
Censored	0	287	491	631	735	789
Events	0					

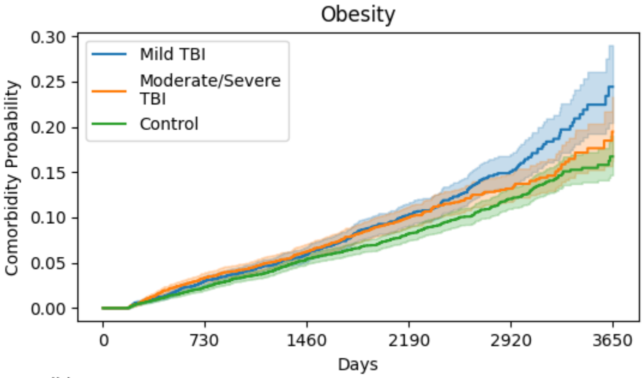

Mild TBI						
At risk	5100	3385	2075	1159	479	67
Censored	0	1590	2819	3658	4294	4679
Events	0	125	206	283	327	354
Moderate/Severe						
TBI		3424	2201	1373	659	78
At risk	5100	1532	2673	3429	4105	4665
Censored	0	144	226	298	336	357
Events	0					
Control		6863	4307	2597	1167	160
At risk	10200	3143	5523	7132	8485	9459
Censored	0	194	370	471	548	581
Events	0					

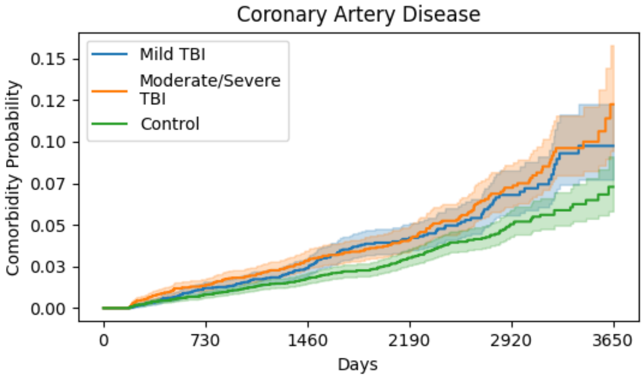

Mild TBI						
At risk	5100	3437	2152	1235	531	84
Censored	0	1613	2866	3750	4431	4868
Events	0	50	82	115	138	148
Moderate/Severe						
TBI		3488	2268	1460	718	95
At risk	5100	1552	2729	3513	4219	4825
Censored	0	60	103	127	163	180
Events	0					
Control		6942	4425	2679	1224	174
At risk	10200	3189	5652	7354	8770	9807
Censored	0	69	123	167	206	219
Events	0					

eFigure 1c, Kaplan-Meier Curves, Cardiovascular Disorders.

© 2024 Halabi C et al. *JAMA Network Open.*

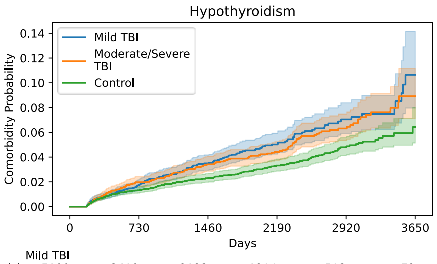

**Mild TBI**						
At risk	5100	3419	2123	1214	513	72
Censored	0	1606	2851	3733	4415	4849
Events	0	75	126	153	172	179
**Moderate/Severe TBI**						
At risk	5100	3458	2238	1436	703	91
Censored	0	1556	2735	3518	4228	4829
Events	0	86	127	146	169	180
**Control**						
At risk	10200	6907	4398	2658	1212	169
Censored	0	3175	5627	7331	8749	9782
Events	0	118	175	211	239	249

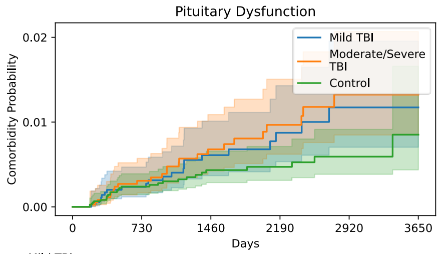

**Mild TBI**						
At risk	5100	3475	2193	1285	563	96
Censored	0	1617	2891	3796	4516	4983
Events	0	8	16	19	21	21
**Moderate/Severe TBI**						
At risk	5100	3526	2315	1502	757	107
Censored	0	1564	2767	3576	4318	4968
Events	0	10	18	22	25	25
**Control**						
At risk	10200	6976	4483	2742	1276	186
Censored	0	3208	5693	7433	8897	9986
Events	0	16	24	25	27	28

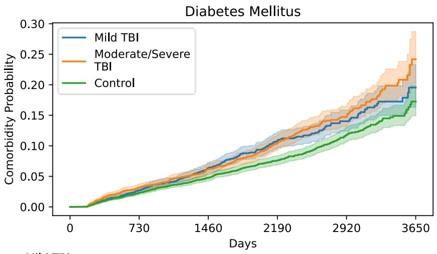

**Mild TBI**						
At risk	5100	3384	2060	1139	469	72
Censored	0	1600	2825	3670	4312	4693
Events	0	116	215	291	319	335
**Moderate/Severe TBI**						
At risk	5100	3417	2167	1339	630	73
Censored	0	1545	2710	3461	4119	4644
Events	0	138	223	300	351	383
**Control**						
At risk	10200	6845	4305	2569	1144	148
Censored	0	3158	5558	7190	8535	9498
Events	0	197	337	441	521	554

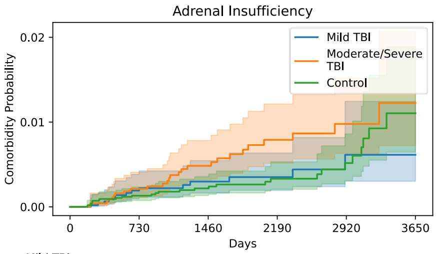

**Mild TBI**						
At risk	5100	3472	2196	1286	563	96
Censored	0	1619	2893	3802	4523	4990
Events	0	9	11	12	14	14
**Moderate/Severe TBI**						
At risk	5100	3528	2317	1502	758	108
Censored	0	1563	2766	3575	4317	4966
Events	0	9	17	23	25	26
**Control**						
At risk	10200	6979	4485	2740	1272	186
Censored	0	3209	5698	7439	8904	9985
Events	0	12	17	21	24	29

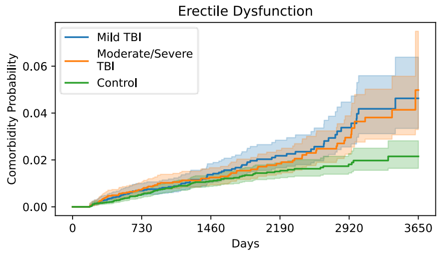

**Mild TBI**						
At risk	5100	3450	2169	1261	543	91
Censored	0	1620	2883	3777	4485	4932
Events	0	30	48	62	72	77
**Moderate/Severe TBI**						
At risk	5100	3505	2293	1477	736	101
Censored	0	1562	2759	3564	4293	4920
Events	0	33	48	59	71	79
**Control**						
At risk	10200	6946	4444	2707	1252	182
Censored	0	3209	5678	7400	8849	9916
Events	0	45	78	93	99	102

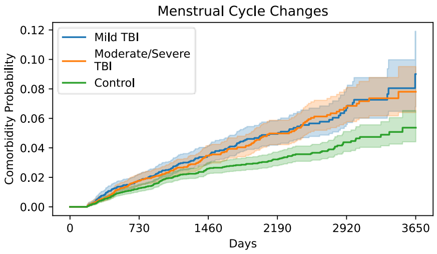

**Mild TBI**						
At risk	5100	3416	2133	1235	535	91
Censored	0	1607	2842	3713	4398	4836
Events	0	77	125	152	167	173
**Moderate/Severe TBI**						
At risk	5100	3472	2238	1419	691	96
Censored	0	1553	2739	3530	4238	4829
Events	0	75	123	151	171	175
**Control**						
At risk	10200	6903	4392	2678	1226	183
Censored	0	3189	5624	7316	8746	9782
Events	0	108	184	206	228	235

eFigure 1d, Kaplan-Meier Curves, Endocrine Disorders.

© 2024 Halabi C et al. *JAMA Network Open.*

	Age Group	18-40		41-60		61-90	
	TBI Severity	mTBI	msTBI	mTBI	msTBI	mTBI	msTBI
Category	Diagnosis						
Neurological disorders	Dementia	10.27 (4.08 - 25.87)*	7.67 (2.99 - 19.68)*	3.88 (2.20 - 6.85)*	2.58 (1.42 - 4.71)*	3.50 (2.46 - 4.98)*	2.88 (2.01 - 4.12)*
	Ischemic stroke/TIA	1.50 (0.76 - 2.97)	1.53 (0.79 - 2.99)	1.61 (1.06 - 2.45)	1.70 (1.13 - 2.55)	2.22 (1.51 - 3.27)*	2.68 (1.85 - 3.87)*
	Seizure disorder	2.43 (1.77 - 3.34)*	2.80 (2.06 - 3.79)*	4.35 (2.88 - 6.57)*	3.89 (2.56 - 5.90)*	5.13 (2.28 - 11.55)*	6.83 (3.15 - 14.84)*
Psychiatric disorders	Anxiety disorder	1.97 (1.73 - 2.25)*	1.84 (1.62 - 2.10)*	2.43 (2.02 - 2.93)*	2.18 (1.81 - 2.64)*	2.96 (2.17 - 4.03)*	2.91 (2.13 - 3.97)*
	Depression	2.45 (2.11 - 2.84)*	2.13 (1.83 - 2.48)*	2.84 (2.33 - 3.47)*	2.25 (1.83 - 2.76)*	3.27 (2.42 - 4.42)*	2.71 (1.98 - 3.70)*
	Bipolar disorder	2.32 (1.60 - 3.37)*	2.56 (1.79 - 3.67)*	3.39 (2.21 - 5.20)*	2.37 (1.50 - 3.74)*	3.71 (1.27 - 10.89)	4.36 (1.54 - 12.35)
	Schizophrenia psychosis	2.43 (1.78 - 3.31)*	3.13 (2.33 - 4.19)*	2.15 (1.45 - 3.17)*	3.34 (2.34 - 4.75)*	4.82 (2.64 - 8.81)*	3.63 (1.94 - 6.79)*
	Suicide ideation intent attempt	2.53 (1.81 - 3.55)*	2.66 (1.92 - 3.69)*	4.84 (3.01 - 7.78)*	4.08 (2.51 - 6.62)*	2.79 (1.23 - 6.33)	2.36 (1.02 - 5.44)
	Sleep disorder	1.86 (1.53 - 2.26)*	1.59 (1.30 - 1.94)*	1.99 (1.63 - 2.42)*	1.45 (1.17 - 1.79)*	2.38 (1.78 - 3.18)*	1.96 (1.45 - 2.64)*
	Alcohol misuse	2.61 (1.90 - 3.59)*	3.12 (2.31 - 4.22)*	2.50 (1.81 - 3.46)*	3.29 (2.44 - 4.44)*	3.42 (1.66 - 7.03)*	3.32 (1.62 - 6.77)*
	Opioid misuse	2.53 (1.68 - 3.82)*	2.87 (1.94 - 4.25)*	3.04 (1.86 - 4.97)*	2.41 (1.44 - 4.04)*	2.31 (0.94 - 5.68)	2.57 (1.07 - 6.19)
	Substance misuse	2.06 (1.67 - 2.54)*	2.25 (1.84 - 2.75)*	2.43 (1.82 - 3.24)*	2.85 (2.16 - 3.77)*	2.71 (1.25 - 5.87)	3.19 (1.50 - 6.79)
Cardiovascular disorders	Coronary artery disease	2.18 (1.15 - 4.11)	1.90 (1.00 - 3.61)	1.25 (0.86 - 1.81)	1.81 (1.30 - 2.51)*	2.45 (1.79 - 3.35)*	2.61 (1.92 - 3.54)*
	Hyperlipidemia	1.15 (0.90 - 1.47)	1.21 (0.95 - 1.54)	1.17 (0.98 - 1.39)	1.07 (0.90 - 1.27)	1.70 (1.40 - 2.08)*	1.99 (1.64 - 2.41)*
	Hypertension	1.10 (0.87 - 1.40)	1.03 (0.81 - 1.30)	1.21 (1.02 - 1.43)	1.44 (1.23 - 1.68)*	1.77 (1.48 - 2.11)*	1.67 (1.40 - 1.98)*
	Obesity	1.08 (0.88 - 1.32)	1.15 (0.94 - 1.39)	1.33 (1.07 - 1.66)	1.35 (1.08 - 1.68)	2.67 (1.90 - 3.75)*	2.45 (1.74 - 3.46)*
Endocrine disorders	Adrenal insufficiency	0.81 (0.18 - 3.60)	4.12 (1.51 - 11.22)	2.77 (0.83 - 9.31)	4.91 (1.65 - 14.63)	1.01 (0.30 - 3.45)	0.94 (0.29 - 3.10)
	Diabetes mellitus	1.04 (0.78 - 1.38)	1.06 (0.80 - 1.40)	1.19 (0.97 - 1.45)	1.33 (1.09 - 1.61)	1.74 (1.36 - 2.22)*	1.90 (1.50 - 2.41)*
	Hypothyroidism	0.76 (0.50 - 1.16)	1.13 (0.79 - 1.63)	1.27 (0.93 - 1.72)	1.26 (0.93 - 1.69)	1.81 (1.33 - 2.46)*	1.64 (1.20 - 2.23)*
	Pituitary dysfunction	1.51 (0.68 - 3.35)	1.72 (0.82 - 3.62)	1.31 (0.39 - 4.34)	6.09 (2.43 - 15.24)*	1.30 (0.37 - 4.54)	0.65 (0.14 - 3.14)
	Erectile dysfunction	2.59 (1.54 - 4.36)*	3.30 (1.93 - 5.67)*	1.28 (0.82 - 1.99)	1.22 (0.78 - 1.91)	1.70 (0.84 - 3.45)	1.62 (0.80 - 3.29)
	Menstrual cycle changes	1.44 (1.16 - 1.80)*	1.30 (1.04 - 1.62)	1.33 (0.86 - 2.06)	1.72 (1.16 - 2.57)	0.06 (0.00 - 6.86)	1.49 (0.25 - 8.94)

eTable 2. Cox Proportional Hazard Ratios Stratified by Age. Age groups include young (18-40 years old), middle-age (41-60), and older (61-90) adults. Asterisks and colored boxes denote significance ($p < 0.002$).

© 2024 Halabi C et al. *JAMA Network Open.*

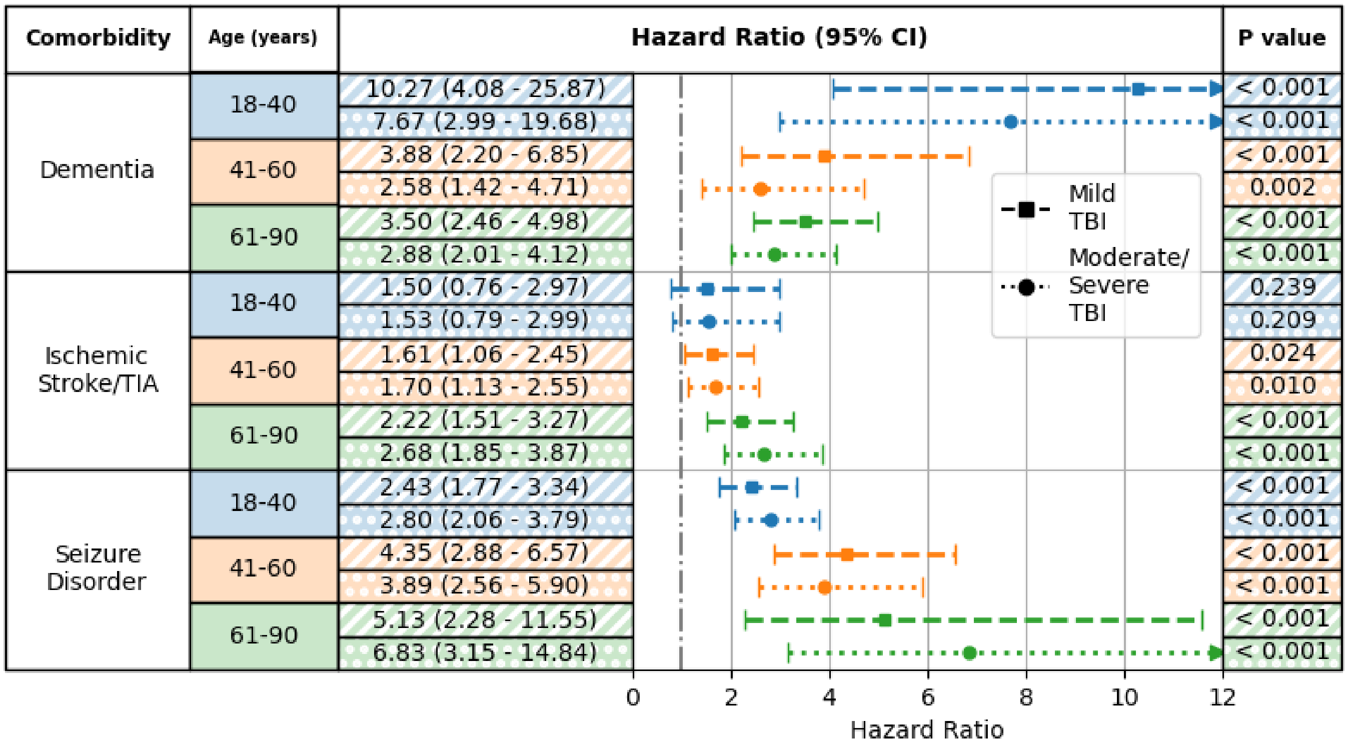

eFigure 2a. Cox Proportional Hazard Ratios Stratified by Age, Neurological Whisker Plots.

© 2024 Halabi C et al. *JAMA Network Open.*

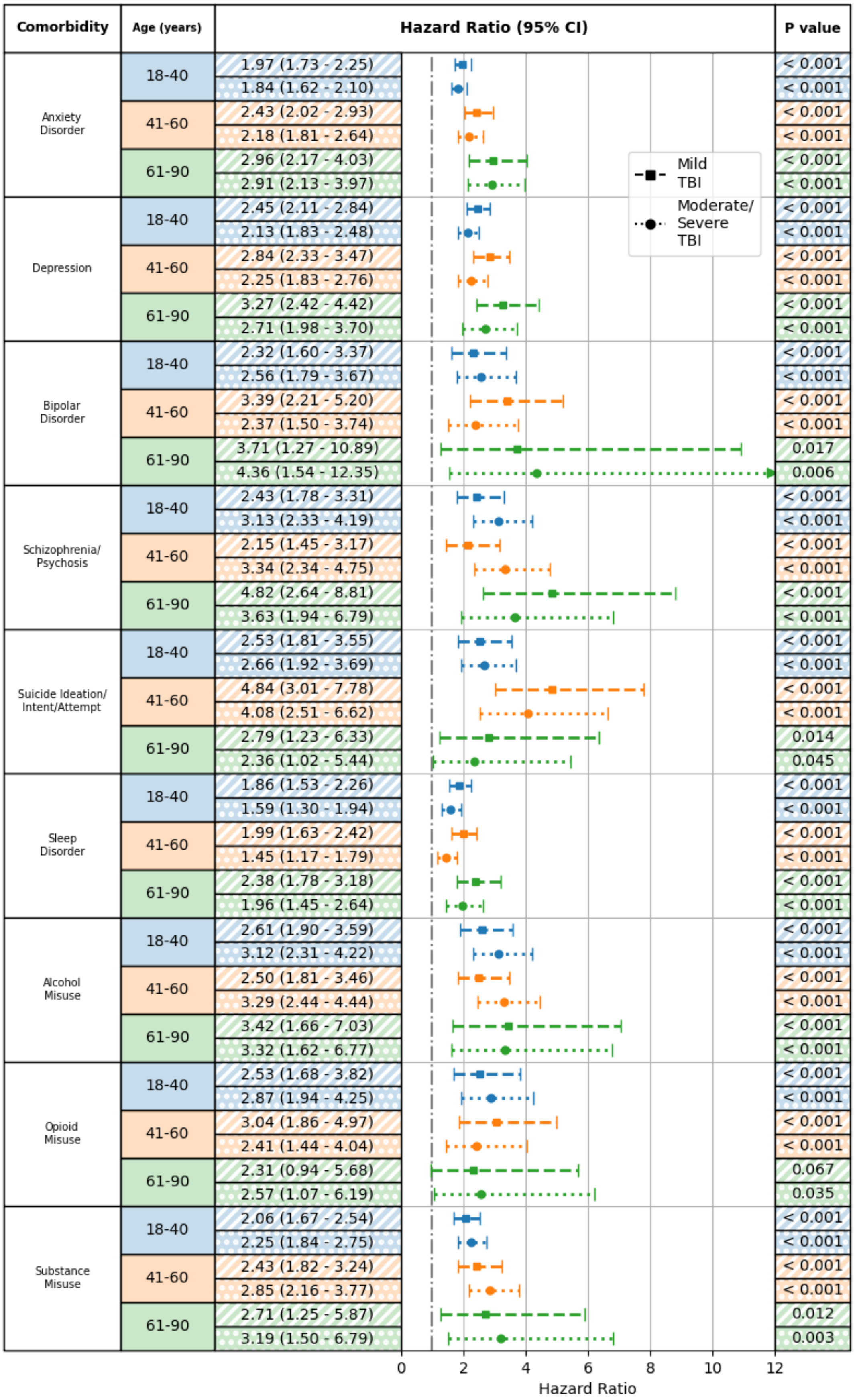

eFigure 2b. Cox Proportional Hazard Ratios Stratified by Age, Psychiatric Whisker Plots.

© 2024 Halabi C et al. *JAMA Network Open.*

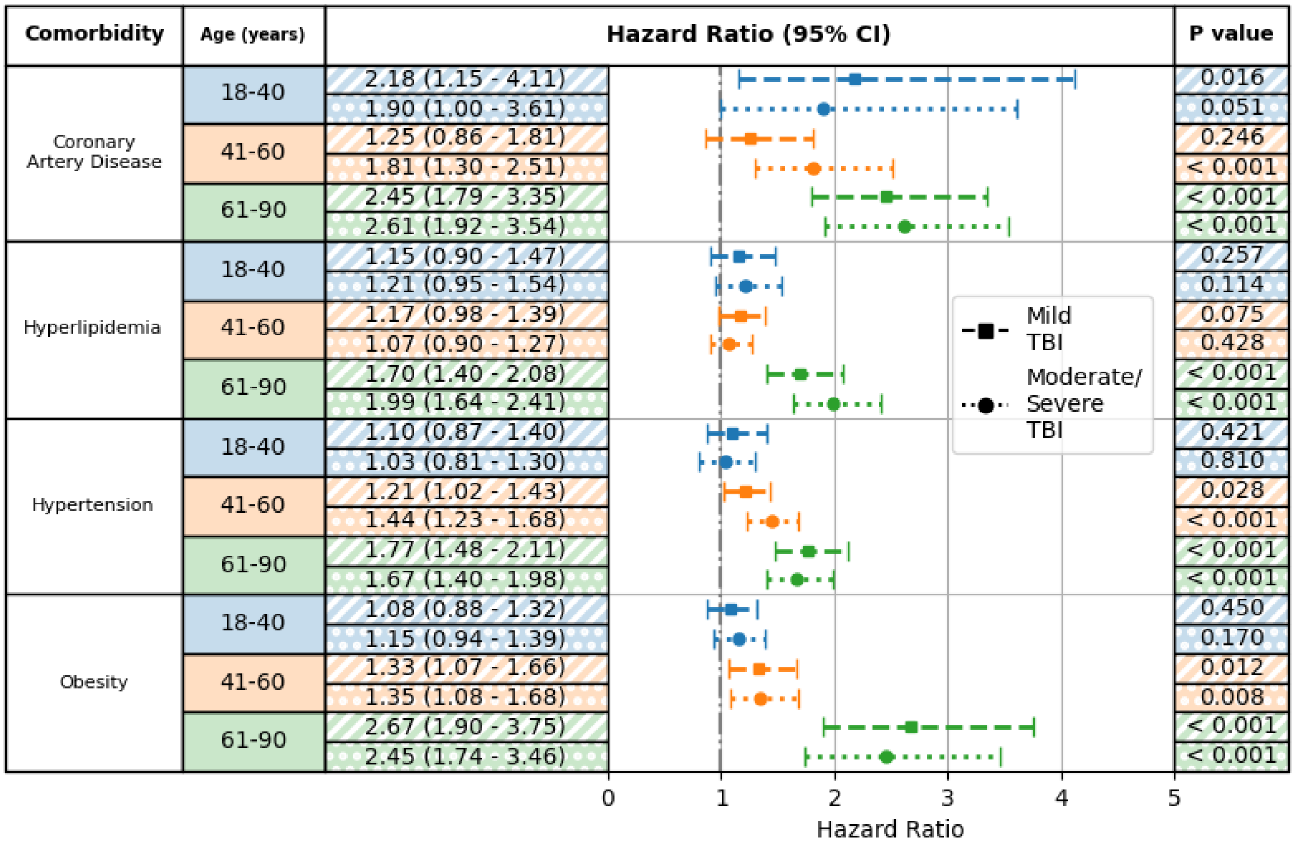

Comorbidity	Age (years)	Hazard Ratio (95% CI)	P value
Coronary Artery Disease	18-40	2.18 (1.15 - 4.11)	0.016
		1.90 (1.00 - 3.61)	0.051
	41-60	1.25 (0.86 - 1.81)	0.246
		1.81 (1.30 - 2.51)	< 0.001
	61-90	2.45 (1.79 - 3.35)	< 0.001
		2.61 (1.92 - 3.54)	< 0.001
Hyperlipidemia	18-40	1.15 (0.90 - 1.47)	0.257
		1.21 (0.95 - 1.54)	0.114
	41-60	1.17 (0.98 - 1.39)	0.075
		1.07 (0.90 - 1.27)	0.428
	61-90	1.70 (1.40 - 2.08)	< 0.001
		1.99 (1.64 - 2.41)	< 0.001
Hypertension	18-40	1.10 (0.87 - 1.40)	0.421
		1.03 (0.81 - 1.30)	0.810
	41-60	1.21 (1.02 - 1.43)	0.028
		1.44 (1.23 - 1.68)	< 0.001
	61-90	1.77 (1.48 - 2.11)	< 0.001
		1.67 (1.40 - 1.98)	< 0.001
Obesity	18-40	1.08 (0.88 - 1.32)	0.450
		1.15 (0.94 - 1.39)	0.170
	41-60	1.33 (1.07 - 1.66)	0.012
		1.35 (1.08 - 1.68)	0.008
	61-90	2.67 (1.90 - 3.75)	< 0.001
		2.45 (1.74 - 3.46)	< 0.001

eFigure 2c. Cox Proportional Hazard Ratios Stratified by Age, Cardiovascular Whisker Plots.

© 2024 Halabi C et al. *JAMA Network Open.*

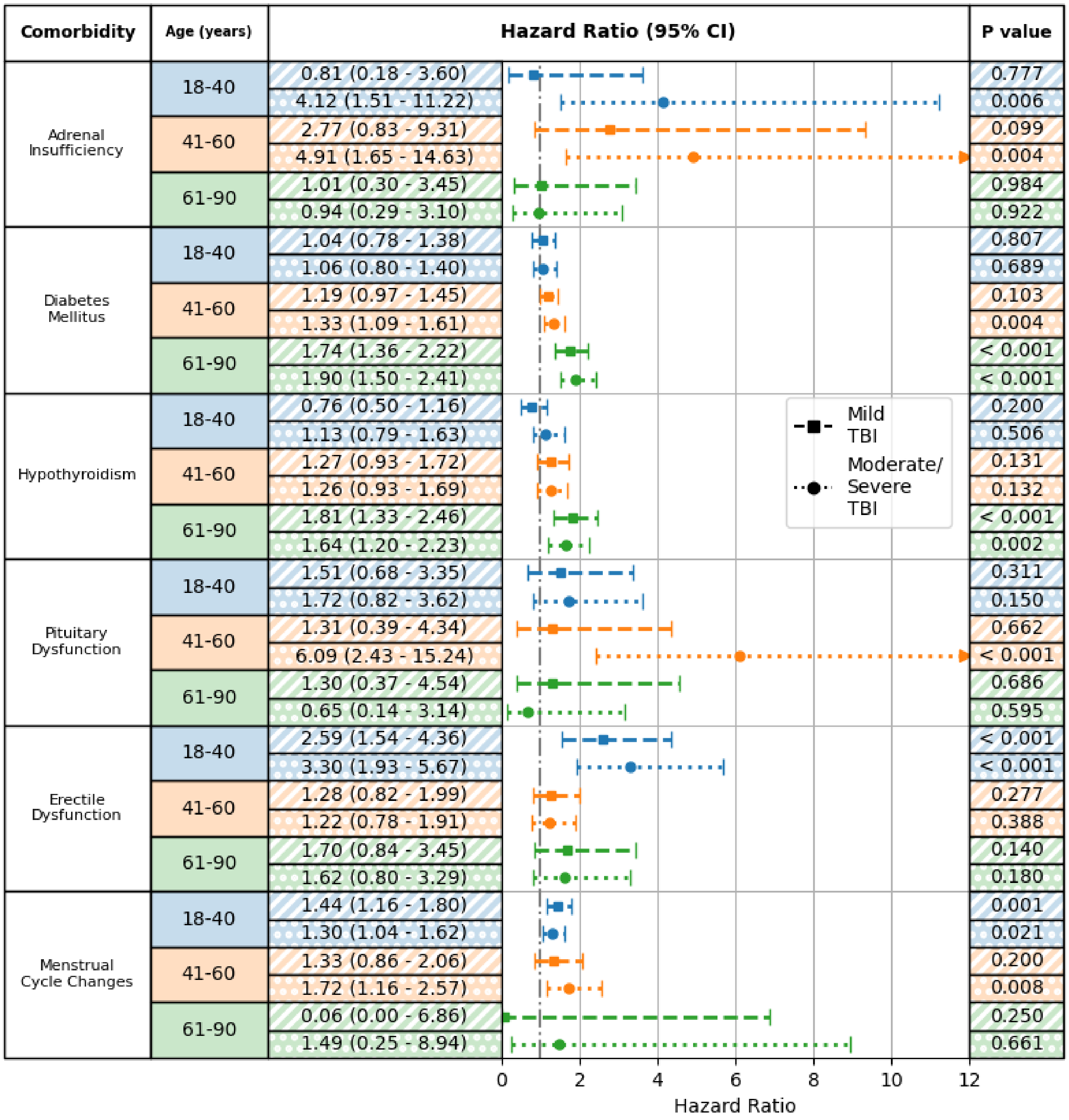

eFigure 2d. Cox Proportional Hazard Ratios Stratified by Age, Endocrine Whisker Plots.

© 2024 Halabi C et al. *JAMA Network Open.*

	ADI Quintile	Low ADI (1,2)		High ADI (9,10)	
	TBI Severity	mTBI	msTBI	mTBI	msTBI
Category	Diagnosis				
Neurological disorders	Dementia	5.04 (3.01 - 8.45)*	2.75 (1.57 - 4.81)*	3.62 (1.49 - 8.78)	6.77 (2.98 - 15.37)*
	Ischemic stroke/TIA	2.05 (1.20 - 3.51)	3.02 (1.86 - 4.89)*	2.28 (1.24 - 4.20)	1.68 (0.89 - 3.17)
	Seizure disorder	2.53 (1.48 - 4.34)*	2.55 (1.50 - 4.33)*	2.36 (1.38 - 4.05)*	3.16 (1.91 - 5.24)*
Psychiatric disorders	Anxiety disorder	2.52 (2.09 - 3.03)*	2.13 (1.76 - 2.57)*	1.57 (1.16 - 2.14)	1.91 (1.43 - 2.54)*
	Depression	3.44 (2.78 - 4.26)*	2.68 (2.15 - 3.35)*	2.47 (1.80 - 3.38)*	2.09 (1.51 - 2.89)*
	Bipolar disorder	1.89 (0.92 - 3.87)	2.03 (1.01 - 4.07)	2.73 (1.50 - 4.98)*	2.15 (1.17 - 3.96)
	Schizophrenia psychosis	4.51 (2.51 - 8.12)*	4.68 (2.63 - 8.34)*	1.76 (1.06 - 2.91)	2.53 (1.61 - 3.97)*
	Suicide ideation intent attempt	3.01 (1.48 - 6.11)	2.85 (1.43 - 5.68)	2.23 (1.36 - 3.66)*	2.06 (1.27 - 3.34)
	Sleep disorder	2.13 (1.71 - 2.65)*	1.56 (1.24 - 1.97)*	1.76 (1.14 - 2.71)	1.65 (1.06 - 2.56)
	Alcohol misuse	4.12 (2.40 - 7.08)*	3.66 (2.13 - 6.27)*	3.02 (1.95 - 4.68)*	3.51 (2.33 - 5.29)*
	Opioid misuse	2.27 (0.92 - 5.61)	2.03 (0.84 - 4.92)	3.54 (1.88 - 6.68)*	3.81 (2.04 - 7.11)*
	Substance misuse	3.27 (1.84 - 5.83)*	3.31 (1.89 - 5.78)*	2.00 (1.46 - 2.74)*	2.12 (1.57 - 2.87)*
Cardiovascular disorders	Coronary artery disease	2.55 (1.65 - 3.94)*	2.39 (1.55 - 3.66)*	1.75 (1.06 - 2.88)	2.39 (1.51 - 3.77)*
	Hyperlipidemia	1.62 (1.34 - 1.98)*	1.68 (1.39 - 2.03)*	1.56 (1.06 - 2.28)	1.36 (0.93 - 2.00)
	Hypertension	1.83 (1.48 - 2.27)*	1.61 (1.30 - 1.99)*	1.27 (0.97 - 1.65)	1.25 (0.96 - 1.62)
	Obesity	1.48 (1.11 - 1.96)	1.49 (1.12 - 1.96)	1.09 (0.75 - 1.58)	1.27 (0.89 - 1.81)
Endocrine disorders	Adrenal insufficiency	1.03 (0.24 - 4.31)	2.43 (0.76 - 7.74)	0.63 (0.06 - 6.09)	2.66 (0.59 - 12.05)
	Diabetes mellitus	1.60 (1.24 - 2.07)*	1.61 (1.26 - 2.06)*	1.05 (0.71 - 1.56)	1.48 (1.04 - 2.09)
	Hypothyroidism	1.42 (1.02 - 1.97)	1.26 (0.91 - 1.75)	1.20 (0.65 - 2.19)	1.45 (0.81 - 2.61)
	Pituitary dysfunction	1.27 (0.55 - 2.94)	0.66 (0.24 - 1.84)	1.99 (0.28 - 14.21)	4.31 (0.78 - 23.84)
	Erectile dysfunction	1.86 (1.11 - 3.10)	1.96 (1.20 - 3.21)	2.11 (0.63 - 7.04)	2.00 (0.61 - 6.62)
	Menstrual cycle changes	1.50 (1.10 - 2.04)	1.41 (1.04 - 1.93)	1.83 (0.91 - 3.70)	1.22 (0.58 - 2.57)

eTable 3. Cox Proportional Hazards Model Stratified by Low and High ADI Quintiles.

© 2024 Halabi C et al. *JAMA Network Open.*

Comorbidity	ADI	Hazard Ratio (95% CI)	P value
Dementia	Low (1,2)	5.04 (3.01 - 8.45)	< 0.001
		2.75 (1.57 - 4.81)	< 0.001
	High (9,10)	3.62 (1.49 - 8.78)	0.004
		6.77 (2.98 - 15.37)	< 0.001
Ischemic Stroke/TIA	Low (1,2)	2.05 (1.20 - 3.51)	0.009
		3.02 (1.86 - 4.89)	< 0.001
	High (9,10)	2.28 (1.24 - 4.20)	0.008
		1.68 (0.89 - 3.17)	0.111
Seizure Disorder	Low (1,2)	2.53 (1.48 - 4.34)	< 0.001
		2.55 (1.50 - 4.33)	< 0.001
	High (9,10)	2.36 (1.38 - 4.05)	0.002
		3.16 (1.91 - 5.24)	< 0.001
Anxiety Disorder	Low (1,2)	2.52 (2.09 - 3.03)	< 0.001
		2.13 (1.76 - 2.57)	< 0.001
	High (9,10)	1.57 (1.16 - 2.14)	0.004
		1.91 (1.43 - 2.54)	< 0.001
Depression	Low (1,2)	3.44 (2.78 - 4.26)	< 0.001
		2.68 (2.15 - 3.35)	< 0.001
	High (9,10)	2.47 (1.80 - 3.38)	< 0.001
		2.09 (1.51 - 2.89)	< 0.001
Bipolar Disorder	Low (1,2)	1.89 (0.92 - 3.87)	0.084
		2.03 (1.01 - 4.07)	0.046
	High (9,10)	2.73 (1.50 - 4.98)	0.001
		2.15 (1.17 - 3.96)	0.014
Schizophrenia/ Psychosis	Low (1,2)	4.51 (2.51 - 8.12)	< 0.001
		4.68 (2.63 - 8.34)	< 0.001
	High (9,10)	1.76 (1.06 - 2.91)	0.030
		2.53 (1.61 - 3.97)	< 0.001
Suicide Ideation/ Intent/Attempt	Low (1,2)	3.01 (1.48 - 6.11)	0.002
		2.85 (1.43 - 5.68)	0.003
	High (9,10)	2.23 (1.36 - 3.66)	0.001
		2.06 (1.27 - 3.34)	0.003
Sleep Disorder	Low (1,2)	2.13 (1.71 - 2.65)	< 0.001
		1.56 (1.24 - 1.97)	< 0.001
	High (9,10)	1.76 (1.14 - 2.71)	0.011
		1.65 (1.06 - 2.56)	0.025
Alcohol Misuse	Low (1,2)	4.12 (2.40 - 7.08)	< 0.001
		3.66 (2.13 - 6.27)	< 0.001
	High (9,10)	3.02 (1.95 - 4.68)	< 0.001
		3.51 (2.33 - 5.29)	< 0.001
Opioid Misuse	Low (1,2)	2.27 (0.92 - 5.61)	0.076
		2.03 (0.84 - 4.92)	0.117
	High (9,10)	3.54 (1.88 - 6.68)	< 0.001
		3.81 (2.04 - 7.11)	< 0.001
Substance Misuse	Low (1,2)	3.27 (1.84 - 5.83)	< 0.001
		3.31 (1.89 - 5.78)	< 0.001
	High (9,10)	2.00 (1.46 - 2.74)	< 0.001
		2.12 (1.57 - 2.87)	< 0.001
Coronary Artery Disease	Low (1,2)	2.55 (1.65 - 3.94)	< 0.001
		2.39 (1.55 - 3.66)	< 0.001
	High (9,10)	1.75 (1.06 - 2.88)	0.027
		2.39 (1.51 - 3.77)	< 0.001
Hyperlipidemia	Low (1,2)	1.62 (1.34 - 1.98)	< 0.001
		1.68 (1.39 - 2.03)	< 0.001
	High (9,10)	1.56 (1.06 - 2.28)	0.023
		1.36 (0.93 - 2.00)	0.118
Hypertension	Low (1,2)	1.83 (1.48 - 2.27)	< 0.001
		1.61 (1.30 - 1.99)	< 0.001
	High (9,10)	1.27 (0.97 - 1.65)	0.079
		1.25 (0.96 - 1.62)	0.099
Obesity	Low (1,2)	1.48 (1.11 - 1.96)	0.007
		1.49 (1.12 - 1.96)	0.005
	High (9,10)	1.09 (0.75 - 1.58)	0.667
		1.27 (0.89 - 1.81)	0.189
Adrenal Insufficiency	Low (1,2)	1.03 (0.24 - 4.31)	0.972
		2.43 (0.76 - 7.74)	0.133
	High (9,10)	0.63 (0.06 - 6.09)	0.688
		2.66 (0.59 - 12.05)	0.205
Diabetes Mellitus	Low (1,2)	1.60 (1.24 - 2.07)	< 0.001
		1.61 (1.26 - 2.06)	< 0.001
	High (9,10)	1.05 (0.71 - 1.56)	0.801
		1.48 (1.04 - 2.09)	0.030
Hypothyroidism	Low (1,2)	1.42 (1.02 - 1.97)	0.037
		1.26 (0.91 - 1.75)	0.166
	High (9,10)	1.20 (0.65 - 2.19)	0.564
		1.45 (0.81 - 2.61)	0.210
Pituitary Dysfunction	Low (1,2)	1.27 (0.55 - 2.94)	0.575
		0.66 (0.24 - 1.84)	0.429
	High (9,10)	1.99 (0.28 - 14.21)	0.493
		4.31 (0.78 - 23.84)	0.094
Erectile Dysfunction	Low (1,2)	1.86 (1.11 - 3.10)	0.018
		1.96 (1.20 - 3.21)	0.007
	High (9,10)	2.11 (0.63 - 7.04)	0.225
		2.00 (0.61 - 6.62)	0.255
Menstrual Cycle Changes	Low (1,2)	1.50 (1.10 - 2.04)	0.011
		1.41 (1.04 - 1.93)	0.029
	High (9,10)	1.83 (0.91 - 3.70)	0.092
		1.22 (0.58 - 2.57)	0.610

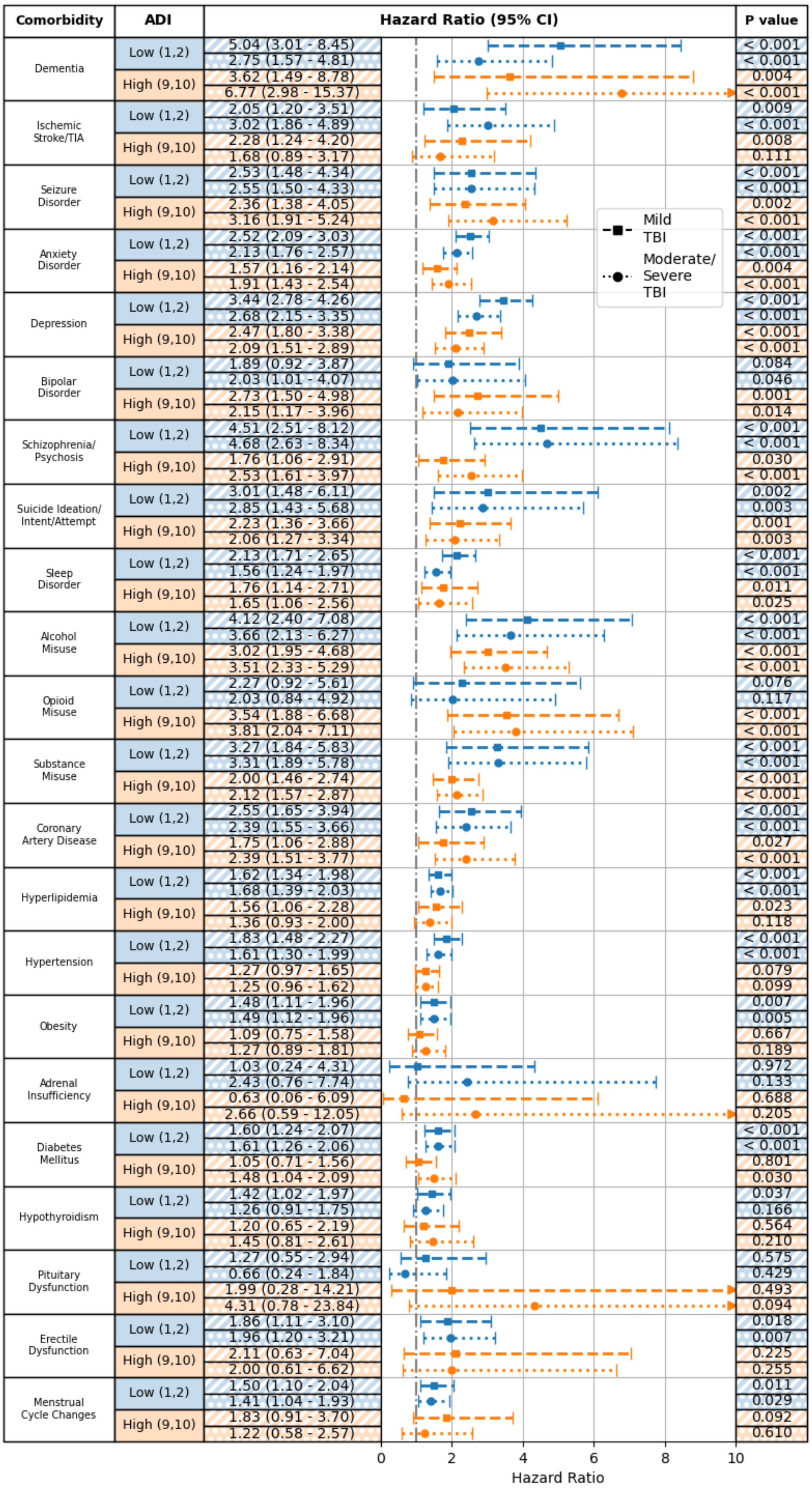

eFigure 3. Cox Proportional Hazards Model Stratified by Low and High ADI Quintiles, Whisker Plots.

© 2024 Halabi C et al. *JAMA Network Open.*

Covariate	Effect Size
Age	0.07
Sex	<0.01
Race/Ethnicity	<0.01
ADI	0.06
Coverage	0.05
Site	<0.01

eFigure 4. Post Matching Effect Sizes.

© 2024 Halabi C et al. *JAMA Network Open.*
