# Supplementary material for: Traumatic Brain Injury and Risk of Incident Comorbidities
Source: JAMA Netw Open. 2024 Dec 12;7(12):e2450499. doi: 10.1001/jamanetworkopen.2024.50499 (PMC11638795; doi:10.1001/jamanetworkopen.2024.50499)
Supplement: Supplement 2. — Data Sharing Statement [file jamanetwopen-e2450499-s002.pdf]

Data Sharing Statement

Halabi. Traumatic Brain Injury and Risk of Incident Comorbidities. *JAMA Netw Open*. Published online December 12, 2024. doi:10.1001/jamanetworkopen.2024.50499

Data
Data available: Yes
Data types: Deidentified participant data, Other (please specify)
Additional Information: Data will be made available to qualified individuals; requests will be subject to review by authors and subject to University of California policies on data sharing.
How to access data: Data will be made available to qualified individuals; requests will be subject to review by authors and subject to University of California policies on data sharing.
When available: With publication

Supporting Documents
Document types: None

Additional Information
Who can access the data: As above; data will be made available to qualified individuals. Requests will be subject to review by authors and subject to University of California policies on data sharing.
Types of analyses: For specific purpose (e.g., external validation, sensitivity analyses).
Mechanisms of data availability: After approval of proposal and with signed data access agreement.
